# Chemical ligation of oligonucleotides using an electrophilic phosphorothioester

**DOI:** 10.1093/nar/gkx459

**Published:** 2017-05-18

**Authors:** Hideto Maruyama, Ryota Oikawa, Mayu Hayakawa, Shono Takamori, Yasuaki Kimura, Naoko Abe, Genichiro Tsuji, Akira Matsuda, Satoshi Shuto, Yoshihiro Ito, Hiroshi Abe

**Affiliations:** 1 Department of Chemistry, Graduate School of Science, Nagoya University, Furo, Chikusa, Nagoya 464-8602, Japan; 2 Faculty of Pharmaceutical Sciences, Hokkaido University, Kita-12, Nishi-6, Kita-ku, Sapporo 060-0812, Japan; 3 Emergent Bioengineering Materials Research Team, RIKEN Center for Emergent Matter Science, 2-1, Hirosawa, Wako-Shi, Saitama, 351-0198, Japan

## Abstract

We developed a new approach for chemical ligation of oligonucleotides using the electrophilic phosphorothioester (EPT) group. A nucleophilic phosphorothioate group on oligonucleotides was converted into the EPT group by treatment with Sanger's reagent (1-fluoro-2,4-dinitrobenzene). EPT oligonucleotides can be isolated, stored frozen, and used for the ligation reaction. The reaction of the EPT oligonucleotide and an amino-modified oligonucleotide took place without any extra reagents at pH 7.0–8.0 at room temperature, and resulted in a ligation product with a phosphoramidate bond with a 39–85% yield. This method has potential uses in biotechnology and chemical biology.

## INTRODUCTION

**Figure 1.**
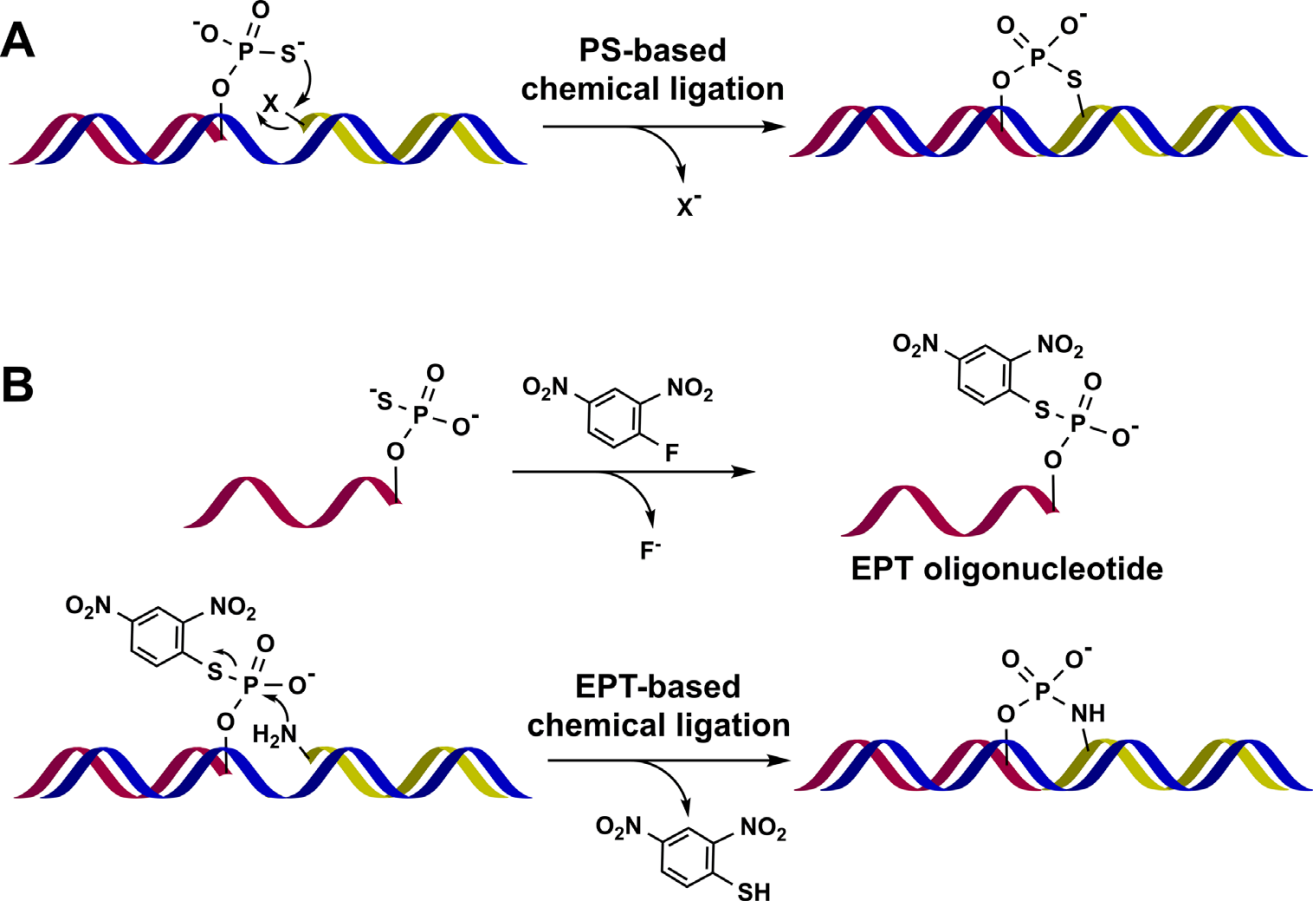
Methods for oligonucleotide chemical ligation. (**A**) Phosphorothioate (PS)-based chemical ligation. (**B**) Electrophilic phosphorothioester (EPT)-based chemical ligation.

Chemical ligation enables covalent bond formation between two oligonucleotide (ON) strands in the presence of a template ON (1–4). Various chemical ligation reactions have been applied to non-enzymatic sequencing of ONs (5), ON-based diagnosis (6), biotechnology (2,7) and nanotechnology for making functional nanoconstructs (8–11). In general, the chemical ligation between two functional groups requires the presence of condensing reagents. Since the first chemical ligation of ONs was achieved with a water-soluble carbodiimide (12), various condensing reagents, for example 1-ethyl-3-(3-dimethylaminopropyl) carbodiimide (EDCI) (13,14), cyanogen bromide (15), imidazole derivatives (16,17) and 1-hydroxybenzotriazole (HOAt) (18), have been utilized to activate the phosphate (PO) group (19). Additionally, a variety of functional group pairs, for example, a nucleophilic group and an electrophilic group, or alkyne and azide groups, have been examined for use in chemical ligations with various applications in biology and biotechnology (2,20). ON-templated reactions without using any extra reagents were developed (2,7,21,22), and they are related to the study on the origin of life (23). These types of reactions are especially suitable for chemical ligations in biological contexts such as in cells, for RNA detection (3) or intracellular build-up of siRNA species (4). Various ligation reactions that do not require additional reagents have been reported (2,6,24). The chemistry requires a pair of reactive groups that are stable in aqueous media. Among them, the phosphorothioate (PS) group, which has strong nucleophilicity, has been utilized in nucleophilic substitution reactions with electrophilic groups like haloacetyl (25,26), tosyl (27), iodo (28,29) and a dabsylated leaving group (30) (Figure 1A). The benefits of PS chemistry include the fact that both chemical and enzymatic syntheses can introduce the PS group at the terminal end of the ON and the ligation reaction yields a product that is similar in structure to the natural ON. A few available ligation methods without extra reagents mainly rely on the nucleophilicity of the PS group. (6) However, the lack of reliable chemistry for the ligation limits its biotechnical applications. Therefore, there is a strong need for developing new techniques for ligation reactions.

Peptide ligation using Sanger's reagent (1-fluoro-2,4-dinitrobenzene; DNFB) was reported (31). Here, DNFB activates a thioacid at the carboxyl terminal of the peptide to give a reactive thioester. The active thioester reacts with an amino group at the N-terminal of another peptide. The reaction provides a good yield of ligated peptide without epimerization (31). We thought the nucleophilic PS group is transformed into the electrophilic phosphorothioester (EPT) group through treatment with DNFB (Figure 1B). Herein, we describe a new method for the chemical ligation of ONs, where the reaction between the EPT group and an amino group on the ON strand results in the formation of a phosphoramidate (PN) bond. The PN bond has a similar structure and properties as a natural phosphodiester (PO) bond with sufficient stability for various biological applications, and thus can act as its replacement (32–35). The EPT group can be introduced at either the 3΄ or 5΄ end of the ON allowing it to form a PN bond at two different sites of the phosphorus centre. Herein, we describe the stability and reactivity of the EPT group, and the use of EPT-based chemical ligation with both DNA and RNA strands.

## MATERIALS AND METHODS

### Synthesis of amino-containing nucleoside units

**Chart 1.**
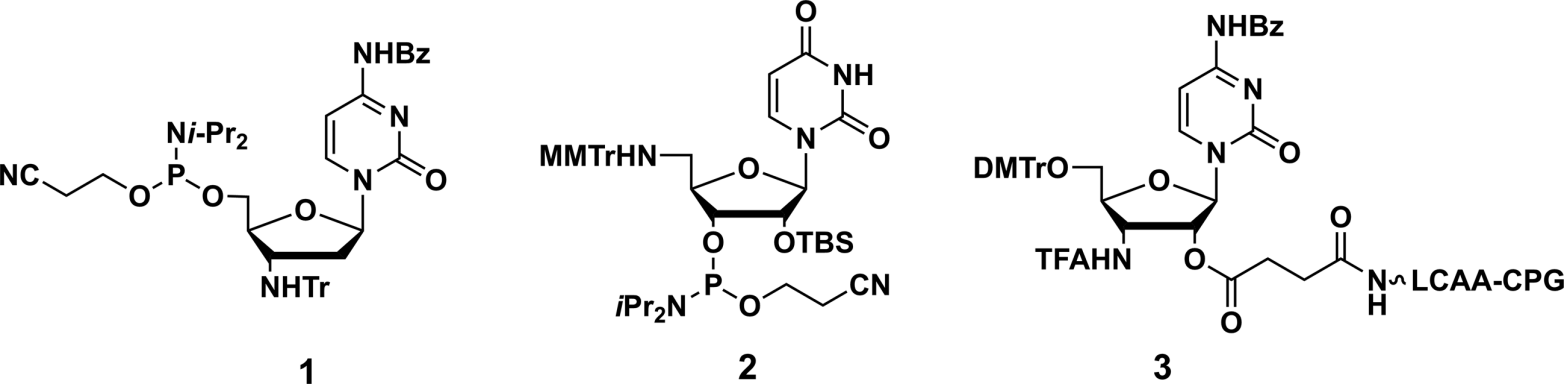
Structure of nucleoside units for the synthesis of amino-modified oligonucleotides.

The synthetic procedures for the preparation of the nucleoside units (**1–3**, Chart 1) and the intermediates (**4–6**) for the synthesis of the amino-modified ONs are provided in the Supplementary Data (Supplementary Schemes S1 and S2).

### Preparation of ONs

DNA and RNA ONs were synthesized on a 0.2 μmol scale using an H-8 SE DNA synthesizer (Gene World) or an NR-2A7MX (Nihon Techno Service) with 2΄-deoxy β-cyanoethyl phosphoramidites (Glen Research) or 2΄-*O*-TOM-protected β-cyanoethyl phosphoramidites (Glen Research), respectively. The DNA ONs were treated with 28% ammonium hydroxide for 10 h at 55°C and concentrated *in vacuo*. Deprotected ONs were purified by 20% denaturing PAGE (7.5 M urea, 1 × TBE), and then isolated by the crush and soak method (elution buffer: 0.2 M NaCl, 10 mM EDTA, pH 8.0). The RNA ONs were treated with AMA reagent (1:1 mixture of 40% aqueous methylamine and 28% ammonium hydroxide) for 10 min at 65°C and concentrated *in vacuo*. Tetra-*n*-butylammonium fluoride (1 M solution in **tetrahydrofuran**) was added to the concentrated residue, which was incubated for 10 min at 55°C, and then at room temperature overnight. The reaction was quenched by the addition of 1 M Tris–HCl (pH 7.2) and desalted with a NAP-25 column (GE Healthcare). Deprotected ONs were purified by PAGE as described above and desalted RNA was precipitated with sodium acetate (pH 5.2) and 2-propanol.

To synthesize a DNA/RNA sequence containing a PS group at the 3΄ end, the first nucleotide was added on a 3΄-phosphate CPG column (Glen Research), followed by sulfurization with a sulfurizing reagent (3*H*-1,2-benzodithiole-3-one-1,1-dioxide, Glen Research), and then the synthesis was carried out according to the normal protocol. To synthesize a DNA/RNA sequence containing a PS group at the 5΄ end, a chemical phosphorylation reagent (Glen Research) was used in the last coupling step, followed by sulfurization with a sulfurizing reagent (3*H*-1,2-benzodithiole-3-one-1,1-dioxide, Glen Research). ONs containing a PS group were used in the subsequent reactions without PAGE purification.

To synthesize a 3΄-NH_2_ DNA sequence containing a fluorescein at the 5΄ end, Universal UnyLinker Support 2000Å (ChemGenes) was coupled with 6-fluorescein phosphoramidite (Glen Research) in the first coupling step, followed by reverse direction ON synthesis with 5΄-CE phosphoramidites (Glen Research). 3΄-Amino dC 5΄-phosphoramidite (**1**) was used in the last coupling step. To synthesize a DNA/RNA sequence containing a fluorescein at the 3΄ end, Universal UnyLinker Support 2000 Å was coupled with 6-fluorescein phosphoramidite (Glen Research) in the first coupling step, and synthesis continued according to the normal protocol. 5΄-Amino-dT-CE phosphoramidite (Glen Research) or 5΄-amino uridine phosphoramidite (**2**) was used in the last coupling step. To synthesize an RNA sequence containing an amino modification at the 3΄ end, the first nucleotide was added on a 3΄-amino CPG (**3**), and 5΄-fluorescein phosphoramidite (Glen Research) was used in the last coupling step to introduce a fluorescein at the 5΄ end.

The ONs containing fluorescein were treated with 28% ammonium hydroxide for 12 h at 55°C and concentrated *in vacuo*. To synthesise the RNA sequence, tetra-*n*-butylammonium fluoride (1 M solution in tetrahydrofuran) was added to the concentrated residue and incubated for 10 min at 55°C, and then at room temperature overnight. The reaction was quenched by the addition of 1 M Tris–HCl (pH 7.2) and desalted by a NAP-25 column (GE Healthcare). Deprotected ONs were purified by reversed-phase HPLC using a Hydrosphere C18 column (10 × 250 mm; YMC) with a trityl group, which was deprotected with aq. 80% AcOH, and the reaction mixture was further purified by reversed-phase HPLC using a Hydrosphere C18 column (4.6 × 250 mm; YMC). Eluent A was 5% acetonitrile (MeCN) in 50 mM triethylammonium acetate (TEAA) buffer (pH 7.0), and eluent B was 100% MeCN. The concentration of eluent B was increased from 10–50% (with trityl group) or 10–20% (without trityl group) over 15 min at a flow rate of 3.0 ml/min (10 × 250 mm; YMC) or 1.0 ml/min (4.6 × 250 mm; YMC). The ON synthesis yields were as follows: 2.8% 3΄-NH_2_ amino DNA, 75% 5΄-PS DNA, 3.2% 5΄-NH_2_ DNA, 42% 3΄-PS DNA, 46% 3΄-NH_2_ RNA, 52% 5΄-PS RNA, 2.3% 5΄-NH_2_ RNA and 57% 2΄-*O*Me-3΄-PS RNA. The purity of the ONs was confirmed by HPLC (Supplementary Figure S9).

### Synthesis of EPT DNA and RNA

Reactions were performed in a 200 μl containing 200 μM of the PS ON and 20 mM 1-fluoro-2,4-dinitrobenzene (Wako Pure Chemical Industries) dissolved in 40% dimethyl sulfoxide containing 20 mM sodium-borate (pH 8.5). The reaction mixture was incubated at room temperature for 2 h and purified by reversed-phase HPLC using a Hydrosphere C18 column (4.6 × 250 mm; YMC). Eluent A was 5% MeCN in 50 mM TEAA buffer (pH 7.0), and eluent B was 100% MeCN. The concentration of eluent B was increased from 10% to 32.5% over 15 min at a flow rate of 1.0 ml/min.

### General procedure of chemical ligation to form a phosphoramidate bond

Reactions were performed in 25 μl containing 4 μM EPT DNA/RNA, 2 μM NH_2_ DNA/RNA and 4 μM template DNA/RNA in 20 mM phosphate buffer (pH 8.0, 7.0 or 6.0) containing 10 mM MgCl_2_ at 25°C. At the appropriate periods, 5 μl aliquots were added to 5 μl of loading buffer (80% formamide, 10 mM EDTA). The reaction was analyzed by electrophoresis on 15% denaturing polyacrylamide gel (PAGE; 5.6 M urea, 25% formamide, 1 × TBE) and visualised by scanning on a BioRad ChemiDoc XRS plus system (BioRad). The chemical ligation yield was determined by the relative fluorescence intensity of the amino-ON and the ligated product. The ligation products isolated by gel extraction after PAGE were analysed by MALDI-TOF MS (Supplementary Table S3), PAGE (Supplementary Figure S10) and HPLC (Supplementary Figure S11).

### Kinetic analysis of chemical ligation

Reactions were performed in 30 μl containing 4 μM PS DNA/RNA, 2 μM NH_2_ DNA/RNA and 4 μM template DNA/RNA in 20 mM phosphate buffer (pH 8.0, 7.0 or 6.0) containing 10 mM MgCl_2_ at 25°C. At the appropriate periods, 5 μl aliquots were added to 5 μl of loading buffer (80% formamide, 10 mM EDTA). The reaction was analysed by electrophoresis on 15% denaturing PAGE (5.6 M urea, 25% formamide, 1 × TBE) and visualised by scanning on a BioRad ChemiDoc XRS plus system (BioRad). C (fluorescein intensity of remaining NH_2_ DNA/RNA)/C_0_ (total fluorescein intensity) versus time was plotted at each sampling time point, and the apparent first-order rate constant (*k*_app_) was analysed by linear regression as simple first-order kinetics by Microsoft Excel.

## RESULTS AND DISCUSSION

### Preparation and Stability Evaluation of EPT-ONs

First, we tested whether the reaction of the PS group and DNFB provides EPT ONs (Supplementary Figure S1). A DNA strand with a PS group at the 5΄ end (5΄-PS DNA) was treated with DNFB in sodium borate buffer (pH 8.5) for 30 min. RP-HPLC analysis showed that the PS-DNA was converted into EPT-DNA, which showed slight absorption at 350 nm, characteristic of the EPT group (36). The MALDI-TOF mass analysis showed only the hydrolysed product, which is DNA with a phosphate group (Supplementary Table S1). A test of the stability of the 3΄-EPT DNA (Supplementary Figure S2) revealed it had a half-life of 8.1 h in pH 7.0 buffer at 25°C and it showed similar stability in pH 8.0 buffer. A solution of 3΄-EPT DNA could be stored without decomposing at −30°C. We also tested the stabilities of 5΄-EPT DNA, 5΄-EPT RNA and 2΄-*O*Me-3΄-EPT RNA (Supplementary Figures S2 and S3). Their half-lives varied from 6.4 to 8.9 h (Supplementary Table S2). We confirmed that the EPT ON was stable in aqueous solution long enough to be isolated and for the ligation reaction to take place. We also investigated 2-chloro-1-methylpyridinium iodide (Mukaiyama reagent) for activation of the PS group. However, the corresponding activated ester was not observed by HPLC analysis. Presumably, it was so reactive that it readily underwent hydrolysis.

### EPT-based DNA ligation

**Figure 2.**
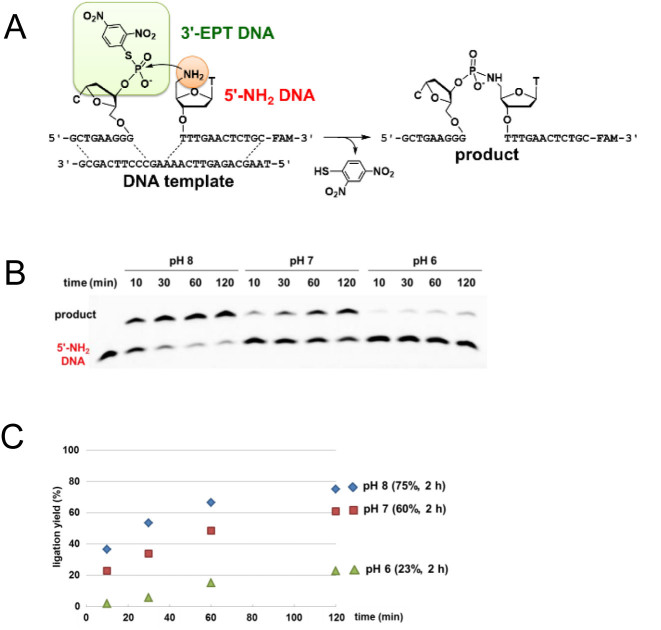
Chemical ligation of 3΄-EPT DNA and 5΄-NH_2_ DNA on template DNA. A solution containing 4 μM EPT DNA, 2 μM 5΄-NH_2_ DNA and 4 μM DNA template was incubated at different pH levels at 25°C. (**A**) Schematic representation of the reaction. (**B**) PAGE analysis (5.6 M urea, 25% formamide, 1 × TBE). The leftmost lane shows the mobility of 5΄-NH_2_ DNA. (**C**) Time course of the ligation yield was determined by the fluorescence intensity of the product. pH 8.0 (diamond), pH 7.0 (square) or pH 6.0 (triangle).

**Figure 3.**
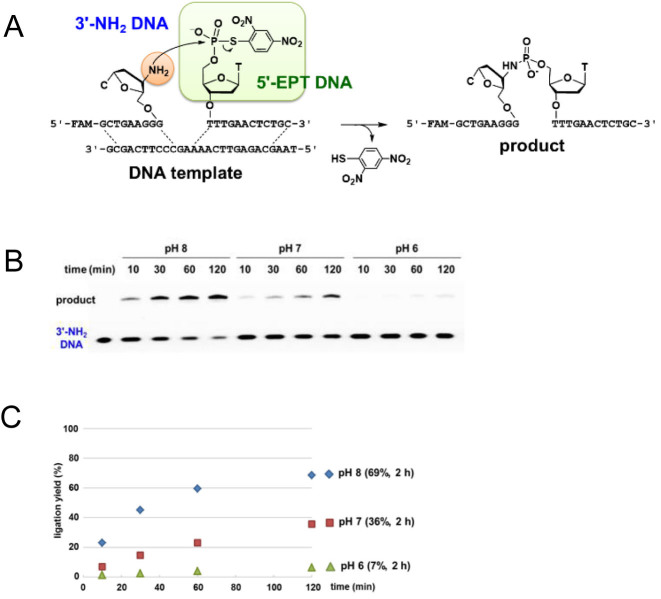
Chemical ligation of 3΄-NH_2_ DNA and 5΄-EPT DNA on a DNA template. The reaction was carried out using 4 μM EPT DNA, 2 μM 3΄-NH_2_ DNA and 4 μM DNA template using similar conditions as in Figure 2. (**A**) Schematic representation of the reaction. (**B**) PAGE analysis. The leftmost lane shows the mobility of 3΄-NH_2_ DNA. (**C**) Time course of the ligation yield. pH 8.0 (diamond), pH 7.0 (square) or pH 6.0 (triangle).

To confirm the reactivity of the EPT group, a DNA-templated reaction between 3΄-EPT DNA and 5΄-amino DNA (5΄-NH_2_ DNA) was carried out (Figure 2A). The template DNA coded a leukemia-related *bcr/abl* gene (37). 3΄-EPT DNA is a 10 mer and 5΄-NH_2_ DNA is a 13 mer with fluorescein (FAM) at the 3΄ terminal. The reactions were performed at pH 6.0, 7.0 and 8.0, and the time course of the reaction was analysed by denaturing PAGE (Figures 2B and 2C). No ligation occurred without the template (Supplementary Figure S8A). In the presence of the DNA template, the reaction at pH 8.0 provided 53% ligation product after 30 min and reached a plateau at 75% product after 120 min. The reaction at pH 7.0 provided 34% ligation product after 30 min and 61% after 120 min. The reaction at pH 6.0 only yielded 23% product after 120 min. The pH dependency of the ligation reaction is presumably related to the protonation of an amino group at the lower pH, which in turn, lowers the reactivity. This experiment confirmed that the EPT-based DNA ligation reaction works at near-neutral pH at room temperature between a pair of 3΄-EPT DNA and 5΄-NH_2_ DNA. To explore the versatility of the EPT-based ligation, we tested the reaction of 5΄-EPT DNA and 3΄-NH_2_ DNA (Figure 3). Reverse-directed ON synthesis was conducted for the preparation of 3΄-NH_2_ DNA using a 3΄-amino dC 5΄-phosphoramidite unit, which was synthesized according to a reported method (Supplementary Scheme S1) (38,39). 5΄-EPT DNA was obtained by treating 5΄-PS DNA with DNFB. The ligation reaction at several pH levels was performed under the same conditions as in Figure 2 and analysed by PAGE (Figures 3B and 3C). The reaction at pH 8.0 had a 45% ligation yield after 30 min and reached a plateau at 69% after 120 min, which is comparable with the reaction of 5΄-NH_2_ DNA. The reactions at pH 7.0 and 6.0 had a 36% and 7% yield, respectively, after 120 min, which is a relatively low yield compared with the reaction of 5΄- NH_2_ DNA.

The equilibrium constants for the formation of the double strands between the 3΄-EPT DNA or 5΄-NH_2_ DNA and the DNA template were calculated as 8.82 × 10^9^ and 10.19 × 10^9^ M^−1^, respectively, based on a thermodynamic study (Supplementary Figure S6). These results indicated that 98.8% of the DNA template formed a ternary complex with 3΄-EPT DNA and 5΄-NH_2_ DNA in the reaction solution at 25°C. Similarly, ternary complex formation of the 5΄-EPT-DNA/3΄-NH_2_ DNA/DNA template was calculated as 98.6% at 25°C. These data suggest that the initial velocity can be analysed as a first-order reaction because of the exclusive presence of the ternary complex. Therefore, the first-order rate constant, *k*, was calculated for the reactions of 3΄-NH_2_ DNA and 5΄-NH_2_ DNA from the results shown in Figures 2 and 3, respectively (Supplementary Figure S4, Table 1). The rate constants for the reactions of 5΄-NH_2_ DNA and 3΄-NH_2_ DNA decreased as the pH dropped from 8.0 to 7.0 in both cases. This can be explained by considering the p*K*_a_ of the amino group. The p*K*_a_ of a 5΄-amino nucleotide and 3΄-amino nucleotide was reported to be 7.8 and 7.7, respectively, which is approximately the same (40,41). The ratio of the unprotonated versus the protonated amino group was calculated from the p*K*_a_ by the Henderson–Hasselbalch equation. The unprotonated form of the 3΄-NH_2_ DNA was calculated to be 67% (pH 8.0), 17% (pH 7.0) and 2% (pH 6.0) of the solution, and that of the 5΄-NH_2_ DNA was calculated to be 61% (pH 8.0), 14% (pH 7.0) and 2% (pH 6.0) of the solution. The percentage of the unprotonated form of the 3΄-NH_2_ DNA or 5΄-NH_2_ DNA was comparable at each pH. At each pH, the rate constant for the reaction between the 5΄-NH_2_ DNA and 3΄-EPT DNA was two times larger compared with that of the reaction between the 3΄-NH_2_ DNA and 5΄-EPT DNA. The difference can be explained by the higher nucleophilicity of the 5΄-NH_2_ group, which is a primary amine.

**Table 1.** The first-order rate constant for the chemical ligation as described in Figures 2–5

	*k* (×10^−2^·min^−1^)			
pH	5΄-NH_2_ DNA	3΄-NH_2_ DNA	5΄-NH_2_ RNA	3΄-NH_2_ RNA
8.0	5.6	2.4	5.0	1.4
7.0	0.95	0.52	1.1	0.76
6.0	0.13	0.064	0.14	0.13

The DNA ligation reaction without FAM labelling was also performed and comparable results were obtained (Supplementary Figures S12–S14). To check whether DNFB reacts with the ONs, causing damage, the template DNA was treated with DNFB and analysed by HPLC and MALDI-MS, and no change was observed (Supplementary Figure S13). The results suggest that DNFB does not react with ONs. Next, the ligation reaction by *in situ* activation was investigated (Supplementary Figure S15). After annealing 5΄-PS DNA, 3΄-amino DNA and the template, the reaction was started by adding DNFB to the solution. The ligation reaction proceeded and gave the product with a 52% yield after 12 h. This ligation method precludes the isolation of EPT-ONs and enables pre-annealing. These features are suitable for ligations with longer ONs that require annealing for double strand formation with the template. Finally, a DNA ligation reaction using longer DNA strands (67 mer 3΄-amino DNA and 45 mer 5΄-EPT DNA) was performed and the ligation yield was 41% after 19 h at pH 8.5 (Supplementary Figures S16 and S17). The 112 mer ligation product was isolated from the PAGE. Sequence analysis was performed to check whether the product was damaged. This analysis clearly showed the formation of the corresponding ligation product (Supplementary Figure S18). The ligated DNA product was amplified by PCR using KOD polymerase. The amplified DNA was inserted into a cloning vector, and 20 clones were obtained. Sequence analysis showed that a single base deletion occurred in two clones out of 20 (Supplementary Figure S18C). It should be noted that no base substitution mutation was detected. This result suggests that treatment with DNFB does not cause severe mutations in the DNA.

### EPT-based RNA ligation

**Figure 4.**
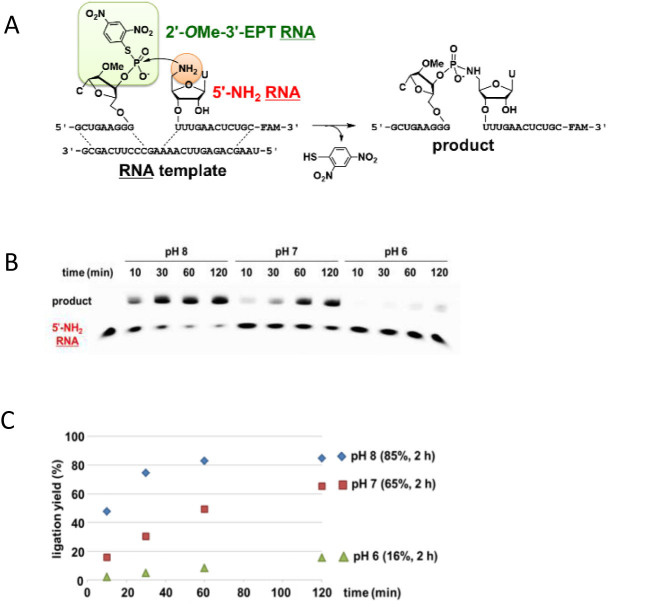
Chemical ligation of 2΄-*O*Me-3΄-EPT RNA and 5΄-NH_2_ RNA on template RNA. The reaction was carried out using 4 μM EPT RNA, 2 μM 5΄-NH_2_ RNA and 4 μM RNA template under similar conditions as in Figure 2. (**A**) Schematic representation of the reaction. (**B**) PAGE analysis. Leftmost lane shows the mobility of 5΄-NH_2_ RNA. (**C**) Time course of the ligation yield. pH 8.0 (diamond), pH 7.0 (square) or pH 6.0 (triangle).

**Figure 5.**
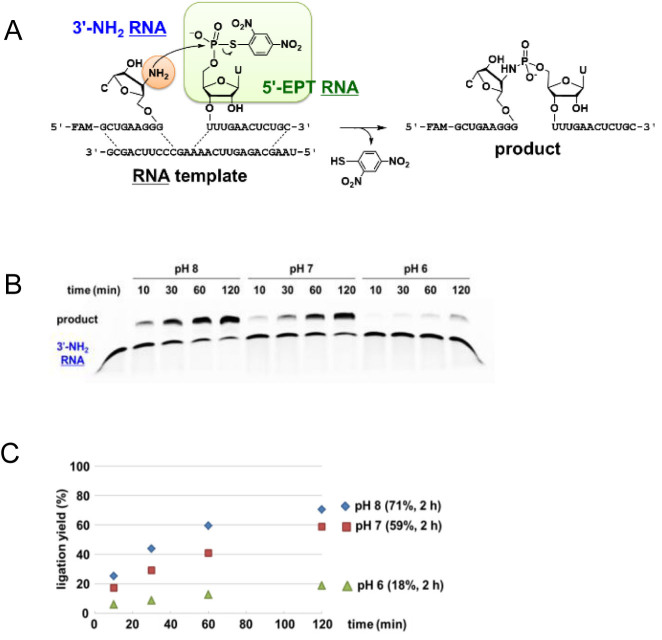
Chemical ligation of 3΄-NH_2_ RNA and 5΄-EPT RNA on template RNA. The reaction was carried out using 4 μM EPT RNA, 2 μM 3΄-NH_2_ RNA and 4 μM RNA template under similar conditions as in Figure 2. (**A**) Schematic representation of the reaction. (**B**) PAGE analysis. Leftmost lane shows the mobility of 3΄-NH_2_ RNA. (**C**) Time course of the ligation yield. pH 8.0 (diamond), pH 7.0 (square) or pH 6.0 (triangle).

Next, we investigated whether EPT ligation can be applied to an RNA strand. The 2΄-*O*Me-3΄-EPT RNA and 5΄-NH_2_ RNA pair (Figure 4), or the 5΄-EPT RNA and 3΄-NH_2_ RNA pair (Figure 5) were designed for the ligation reaction. The RNA template had the identical sequence as the DNA template. The 5΄-deoxy-5΄-aminouridine phosphoramidite unit and the 3΄-deoxy-3΄-aminocytidine CPG unit were synthesised to prepare 5΄-NH_2_ RNA and 3΄-NH_2_ RNA, respectively (Supplementary Schemes S1 and S2). Initially, we used 3΄-EPT RNA for the ligation. However, it was completely converted to cyclic 2΄,3΄-phosphate by an intramolecular reaction of the 3΄-EPT group and the 2΄-hydroxy group. Therefore, 2΄-*O*Me-3΄-EPT RNA was used for the ligation reaction instead of 3΄-EPT RNA.

The reaction of 5΄-NH_2_ RNA and 2΄-*O*Me-3΄-EPT RNA with the RNA template provided a high yield of ligation product, 85% and 65% after 2 h in pH 8.0 and 7.0 buffer, respectively, and a low yield of 16% after 2 h at pH 6.0 (Figure 4). The reaction of 3΄-NH_2_ RNA and 5΄-EPT RNA produced a high yield of ligation product, 71% and 59% after 2 h in pH 8.0 and 7.0 buffer, respectively, and a low yield of 18% after 2 h in pH 6.0 buffer (Figure 5). The reaction yield after 2 h using 5΄-NH_2_ RNA or 3΄-NH_2_ RNA was approximately comparable at each pH. No ligation occurred in the absence of the template (Supplementary Figure S8B).

The equilibrium constants for the formation of the double strands between the EPT RNA or NH_2_ RNA and the RNA template were calculated based on a thermodynamic study (Supplementary Figure S7). From these results, the percentage (%) of ternary complex formation in the reaction solution was calculated to be 99.9% for the 2΄-*O*Me-3΄-EPT RNA/5΄-NH_2_ RNA/RNA template and 99.8% for the 5΄-EPT RNA/3΄-NH_2_ RNA/RNA template. Therefore, the initial velocity of the reaction was determined to be of first order. The first-order rate constant, *k*, was calculated for the RNA ligation (Supplementary Figure S5, Table 1). The *k* for the 5΄-NH_2_ RNA reaction decreased between pH 8.0 and 7.0, and the *k* for the 3΄-NH_2_ RNA reaction decreased between pH 7.0 and 6.0. This can be explained by considering the p*K*_a_ of 5΄-NH_2_ RNA (41) and 3΄-NH_2_ RNA, (41,42) which is expected to be 7.8 and 7.0, respectively. The unprotonated form (%) of 5΄-NH_2_ RNA and 3΄-NH_2_ RNA at pH 8.0 was calculated to be 61% and 91%, respectively. The *k* ratio of 3.6 for 5΄-NH_2_ RNA to 3΄-NH_2_ RNA at pH 8.0 can be explained by their different nucleophilicities.

### Comparison of DNA and RNA ligation

We also examined the difference between DNA and RNA in EPT ligation. The *k* for 5΄-NH_2_ DNA and 5΄-NH_2_ RNA was similar at all pH levels. In contrast, the *k* of the reaction of 3΄-NH_2_ DNA and 3΄-NH_2_ RNA varied depending on the pH. Two factors influenced the reaction outcome of 3΄-NH_2_ ONs. First, the p*K*_a_ of the 3΄-NH_2_ RNA was lower than that of the 3΄-NH_2_ DNA as mentioned previously. Secondly, the nucleophilicity of the 3΄-NH_2_ DNA should be higher than that of the 3΄-NH_2_ RNA because of the steric effect of the 2΄ hydroxy group.

In conclusion, EPT ligation was developed as a useful ligation method. The PS group, which has previously been used as a nucleophilic group, was converted to an electrophilic group (EPT) using Sanger's reagent (DNFB). The method relies on the polarity reversal of the nucleophilic PS group. The method was applied for the ligation of both DNA and RNA strands. The EPT group can be positioned at either the 3΄ or 5΄ terminal of the ON. The reaction takes place at near-neutral pH (7.0 or 8.0) at room temperature, providing a ligation product with a 39%–85% yield. The method has potential uses in biotechnology and chemical biology.

## Supplementary Material

Supplementary Data
