# Supplementary material for: Chemical ligation of oligonucleotides using an electrophilic phosphorothioester
Source: Nucleic Acids Res. 2017 May 18;45(12):7042–8. doi: 10.1093/nar/gkx459 (PMC5499596; doi:10.1093/nar/gkx459)
Supplement: Supplementary Data [file gkx459_supp.pdf]

Supplementary Data

Contents

Table S1. Sequences and MALDI-TOF MS analysis of ONs used ……………….………………………….pS2

Figure S1 HPLC analysis of the reaction of 5'-PS DNA and DNFB....……………………………………….pS3

Figure S2 Stability of EPT DNA under the ligation condition………………………………………………..pS4

Figure S3 Stability of EPT RNA under the ligation condition………………………………………………..pS5

Table S2. The calculated half-life ($t_{1/2}$) of EPT oligonucleotides…………………………………………..pS5

Figure S4 Kinetic analysis of reaction of NH_2 DNA and EPT DNA………………………………………….pS6

Figure S5 Kinetic analysis of reaction of NH_2 RNA and EPT RNA………………………………..……...pS7

Figure S6. Melting temperature measurement of DNA……………………………………………………..pS8

Figure S7. Melting temperature measurement of RNA……………………………………………………..pS9

Figure S8 Chemical ligation reaction without template oligonucleotide…………………………………….pS10

Figure S9 HPLC analysis of the ONs used in this study…………………………………………………..pS11

Table S3 MALDI-TOF MS analysis of ligation products…………………………………………………..pS12

Figure S10 PAGE analysis of ligation products……………………………………………………………….pS13

Figure S11 HPLC analysis of the ligation products ……......…………………………………………………pS14

Figure S12 Ligation with non-fluorescence-labelled amino-DNA without isolation of EPT-DNA…………..pS15

Figure S13 HPLC analysis of DNA template after treatment of DNFB……………………………………pS16

Figure S14 MALDI-MS data and HPLC analysis of substrates and ligation product in Supplementary Data.pS17

Figure S15 DNA ligation after annealing and the following *in-situ* activation of 5'-PS DNA by DNFB...…..pS18

Figure S16 Ligation reaction with long deoxyoligonucleotides……………………………………………...pS19

Figure S17 MALDI-MS and HPLC data of substrates for the ligation with long deoxyoligonucleotides……pS20

Figure S18 Sequence analysis of the ligation product from long deoxyoligonucleotides……………………pS21

Procedures and analytical data for chemical synthesis of nucleotide derivatives………………………..pS22 ~ pS31

Scheme S1 Synthesis of 5'-Amino Uridine Phosphoramidite unit **2**……………...………………………......pS22

Scheme S2 Synthesis of 3'-Amino Cytidine CPG unit **3**…………………………………………………...pS23

References……………………………………………………………………………………………………...pS31

Name	Sequences	Mw (M + H)	
		calcd	found
3'-EPT DNA	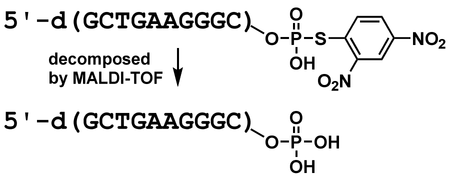	3356.2	-
		3174.0	3175.7
5'-NH_2 DNA	5'-d(NH_2-TTTTGAACTCTGC)-FAM-3'	4483.1	4482.6
5'-EPT DNA	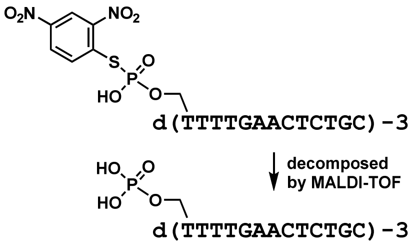	4178.7	-
		3996.5	3995.7
3'-NH_2 DNA	5'-FAM-d(GCTGAAGGGC-NH_2)	3660.5	3660.0
template DNA	5'-d(TAAGCAGAGTTCAAAAGCCCTTCAGCG)-3'	8277.4	8277.1
2'-OMe-3'-EPT RNA	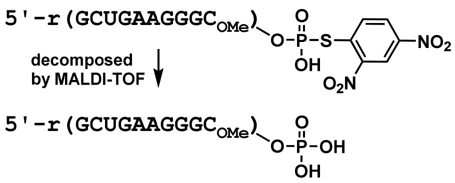	3516.2	-
		3334.0	3336.5
5'-NH_2 RNA	5'-r(NH_2-UUUUGAACUCUGC)-FAM-3'	4606.9	4607.5
5'-EPT RNA	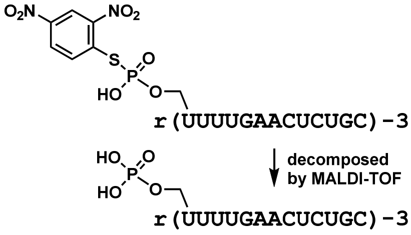	4303.5	-
		4121.4	4121.4
3'-NH_2 RNA	5'-FAM-r(GCUGAAGGGC-NH_2)-3'	3775.5	3779.2
template RNA	5'-r(UAAGCAGAGUUCAAAAGCCCUUCAGCG)-3'	8640.3	8638.4

Table S1 Sequences and MALDI-TOF MS analysis of ONs used in the main text.

Mass data were obtained using a microflex MALDI-TOF mass spectrometer (Bruker Daltonics) by positive m ode using a mixture of 3-hydroxypicolinic acid (HPA) and ammonium citrate as a matrix. EPT DNA/RNA was decomposed to corresponding phosphate DNA/RNA by laser irradiation.

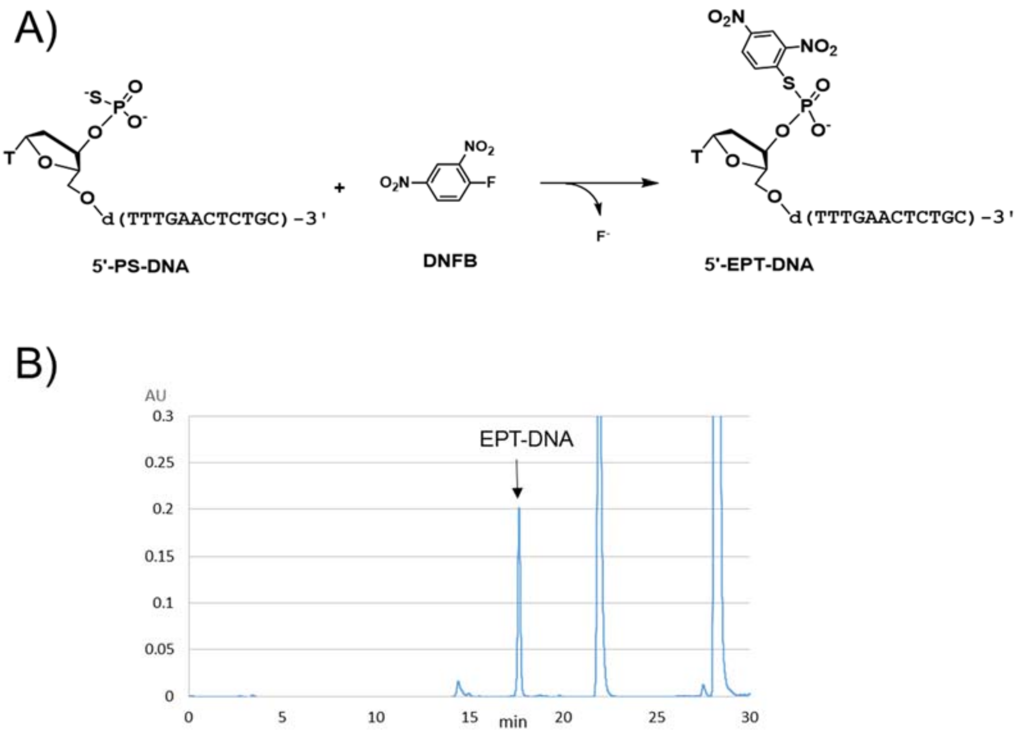

Figure S1. HPLC analysis of the reaction of 5'-PS DNA and DNFB. A) Schematic representation of the reaction of 5'-PS DNA and DNFB. B) RP-HPLC analysis of the reaction. 100 µM of 5′-PS DNA and 40 mM of DNFB in 50 mM sodium tetraborate (pH 8.5) incubated at rt for 30 min. The conversion yield was calculated to be 83% after 30 min based on HPLC analysis of the reaction mixture. HPLC analysis was performed using: hydrosphere C18 column (4.6 x 250 mm; YMC). Eluent A was 5% acetonitrile (MeCN) in 50 mM triethylammonium acetate (TEAA) buffer (pH 7.0), and eluent B was 100% MeCN. The concentration of eluent B was increased from 0–40% over 20 min, at a flow rate of 1.0 mL/min. Absorption was monitored at 260 nm. Peaks with retention time of around 23 and 28 min are those of DNFB-derived species.

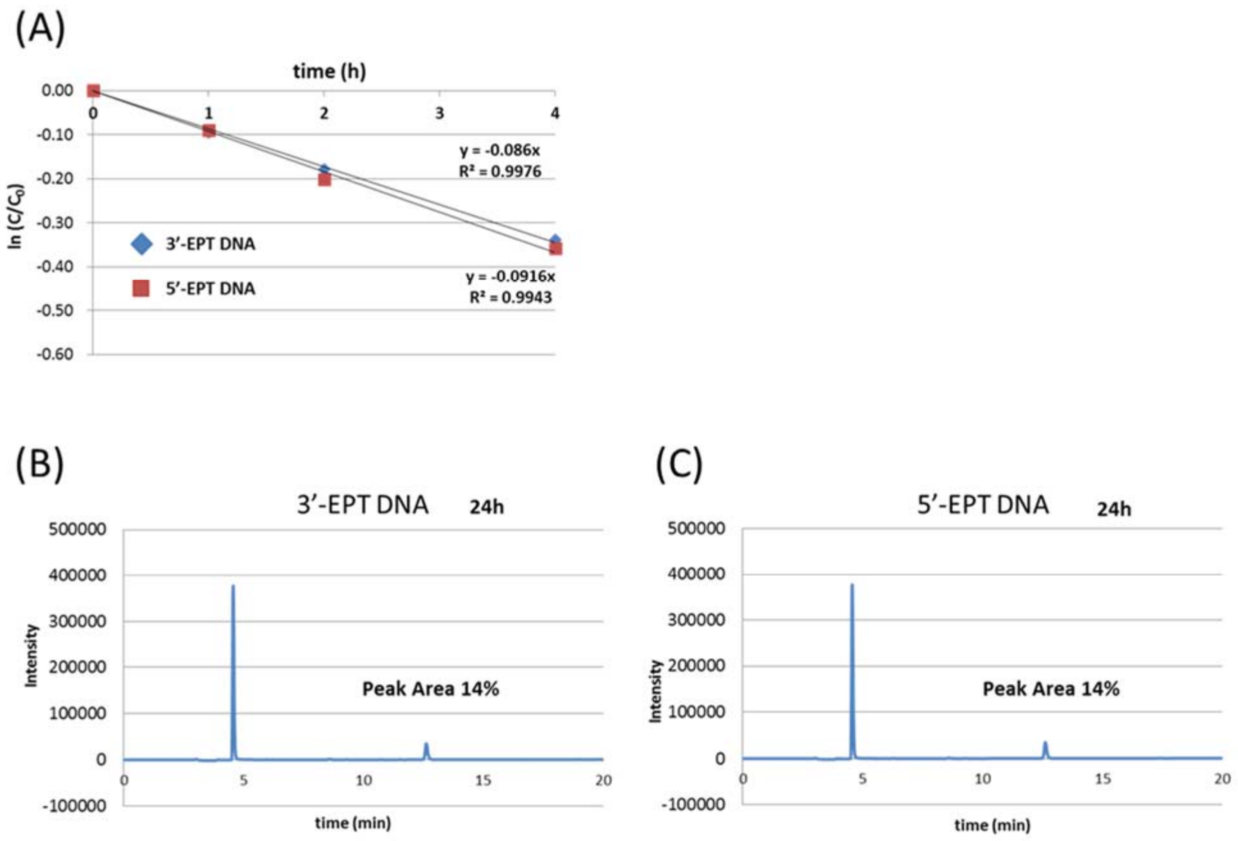

Figure S2 Stability of EPT DNA under the ligation condition. Sequence of 5'-EPT DNA and 3'-EPT DNA is shown in Fig. 2 and 3, respectively. (A) Plots of ln (C/C_0) versus time for the analysis of stability of EPT DNA in the condition of chemical ligation; (B) HPLC analysis of the 3'-EPT DNA after 24h; (C) HPLC analysis of the 5'-EPT DNA after 24 h; Reactions were performed in a 62.5 µL reaction volume, containing 4 µM of the EPT oligonucleotide in 20 mM Phosphate buffer (pH 7.0). The reaction mixture was incubated at 25 °C for 1, 2, 4 h and analysed by reversed-phase HPLC, using a Hydrosphere C18 column (4.6 × 250 mm; YMC). Eluent A was 5% acetonitrile (MeCN) in 50 mM triethylammonium acetate (TEAA) buffer (pH 7.0), and eluent B was 100% MeCN. The concentration of eluent B was increased from 10–32.5% over 15 min, at a flow rate of 1.0 mL/min. Absorption was monitored at 260 nm. ln [C (remaining percentage of EPT DNA/RNA at each sampling point)/C_0 (remaining percentage of EPT DNA/RNA before incubation at 25 °C)] versus time was plotted at each sampling time, and the apparent first-order rate constant (k_{app}) was analyzed by linear regression as simple first-order kinetics by Microsoft Excel.

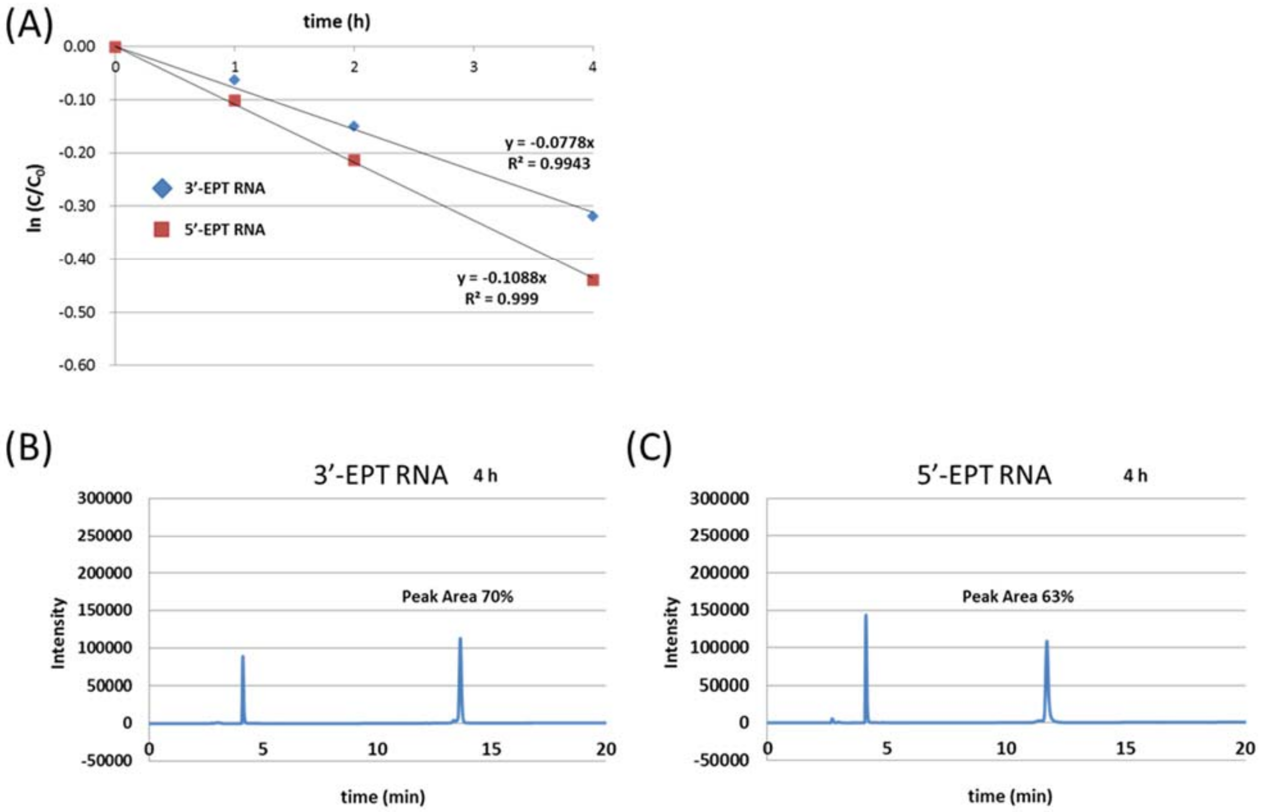

Figure S3 Stability of EPT RNA under the ligation condition. Sequence of 5'-EPT RNA and 3'-EPT RNA is shown in Fig. 4 and 5, respectively. (A) Plots of ln (C/C_0) versus time for the analysis of stability of EPT RNA in the condition of chemical ligation; (B) HPLC analysis of the 3'-EPT RNA after 4h; (C) HPLC analysis of the 5'-EPT RNA after 4h; Reactions were performed in a 62.5 µL reaction volume, containing 4 µM of the EPT oligonucleotide in 20 mM Phosphate buffer (pH 7.0). The reaction mixture was incubated at 25 °C for 1, 2, 4 h and analysed by reversed-phase HPLC, using a Hydrosphere C18 column (4.6 × 250 mm; YMC). Eluent A was 5% acetonitrile (MeCN) in 50 mM triethylammonium acetate (TEAA) buffer (pH 7.0), and eluent B was 100% MeCN. The concentration of eluent B was increased from 10–32.5% over 15 min, at a flow rate of 1.0 mL/min. Absorption was monitored at 260 nm. ln [C (remaining percentage of EPT DNA/RNA at each sampling point)/C_0 (remaining percentage of EPT DNA/RNA before incubation at 25 °C)] versus time was plotted at each sampling time, and the apparent first-order rate constant (k_{app}) was analyzed by linear regression as simple first-order kinetics by Microsoft Excel.

Table S2. The calculated half-life ($t_{1/2}$) of EPT oligonucleotides

	3'-EPT-DNA	5'-EPT-DNA	3'-EPT-RNA	5'-EPT-RNA
$t_{1/2}$ (h)	8.1	9.2	8.9	6.4

(A)

(B)

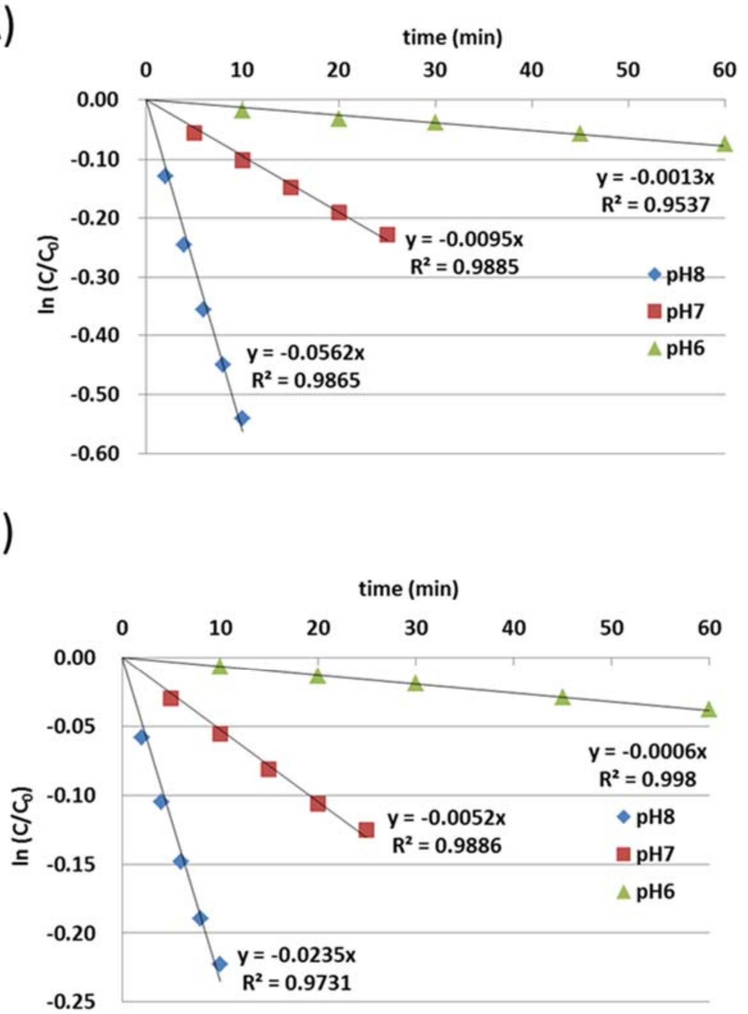

Figure S4 Kinetic analysis of reaction of NH_2 DNA and EPT DNA (A) Plots of ln (C/C_0) versus time in the reaction of 5'- NH_2 DNA and 3'-EPT DNA (B) Plots of ln (C/C_0) versus time in the reaction of 3'-NH_2 DNA 5'-EPT DNA Sampling point: 2, 4, 6, 8, 10 min (pH 8.0; diamond); 5, 10, 15, 20, 25 min (pH 7.0; square); 10, 20, 30, 45, 60 min (pH 6.0, triangle)

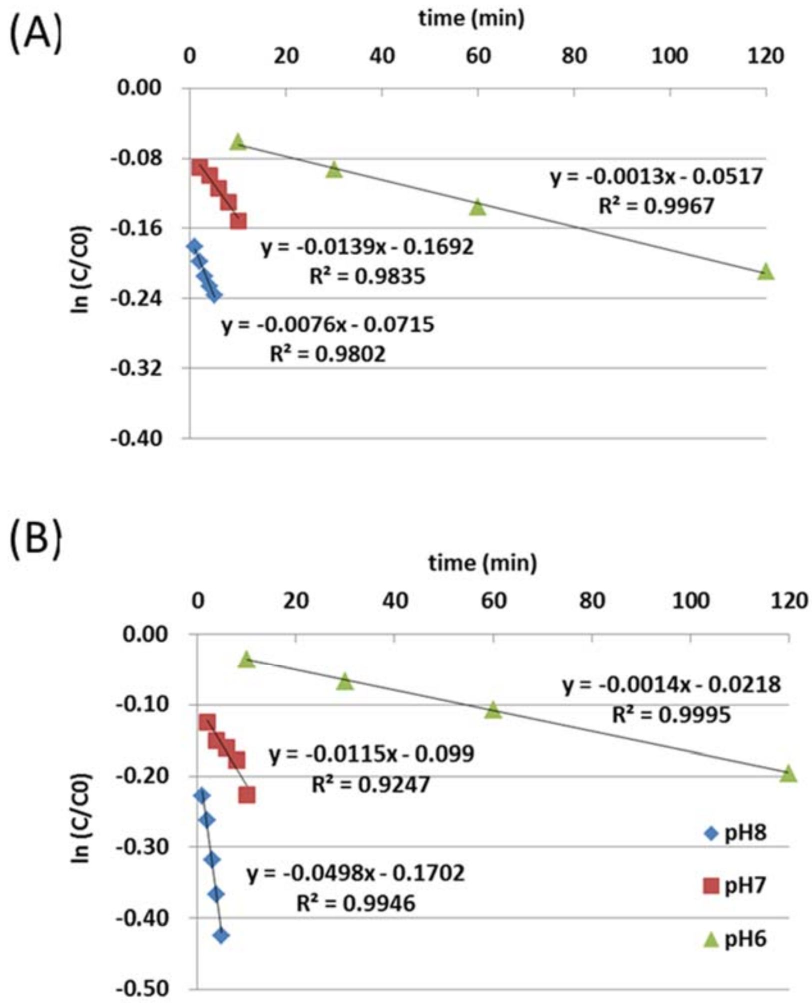

Figure S5 Kinetic analysis of reaction of NH_2 RNA and EPT RNA (A) Plots of ln (C/C_0) versus time in the reaction of 5'- NH_2 RNA and 3'-EPT RNA (B) Plots of ln (C/C_0) versus time in the reaction of 3'- NH_2 RNA and 5'-EPT RNA Complex of NH_2 RNA-EPT RNA-template was not completely denatured in the gel and has the same mobility with ligation product. Thus a line did not cross the origin, however the calculated coefficient of determination (R^2) showed high accuracy (> 0.9). Sampling point: 1, 2, 3, 4, 5 min (pH 8.0; diamond); 2, 4, 6, 8, 10 min (pH 7.0; square); 10, 30, 60, 120 min (pH 6.0, triangle)

(A) Plots of $1/T_m$ versus ln(concentration of duplex/4) of DNA.

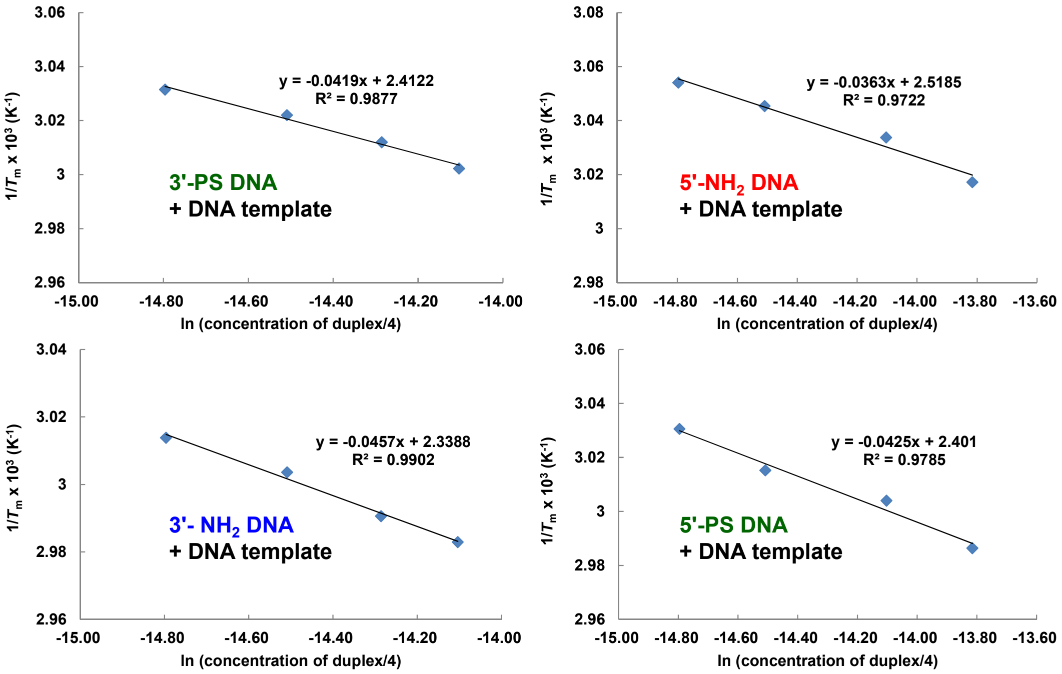

(B) Thermodynamic parameters of DNA.

	ΔH (kJ mol⁻¹)	ΔS (J K⁻¹mol⁻¹)	ΔG_{297} (kJ mol⁻¹)	*K* (x10⁹M⁻¹)	the rate of duplex formation[a]	the rate of ternary complex
3'-PS DNA + DNA template	-198.4	-478.7	-55.8	8.82	99.5	98.8
5'-NH_2 DNA + DNA template	-229.0	-579.1	-56.5	10.19	99.3	
3'- NH_2 DNA + DNA template	-181.9	-425.5	-55.1	6.53	99.1	98.60
5'-PS DNA + DNA template	-195.6	-469.7	-55.7	7.98	99.5	

Figure S6. Melting temperature measurement of DNA. All measurements were done in 20 mM Tris-HCl buffer (pH 7.2) containing 1.5-5 μM duplex and 10 mM $MgCl_2$ in 20 mM HEPES buffer (pH7.0). Reaction was monitored absorbance at 260 nm. (A) Plots of 1/Tm versus ln(concentration of duplex/4). The fitting curve (black line) was analyzed by linear regression as simple first-order kinetics by Microsoft Excel. (B) Thermodynamic parameters. The standard deviations for ΔH and ΔS were estimated from van't Hoff equation and (A).

(A) Plots of $1/T_m$ versus ln(concentration of duplex/4) of RNA.

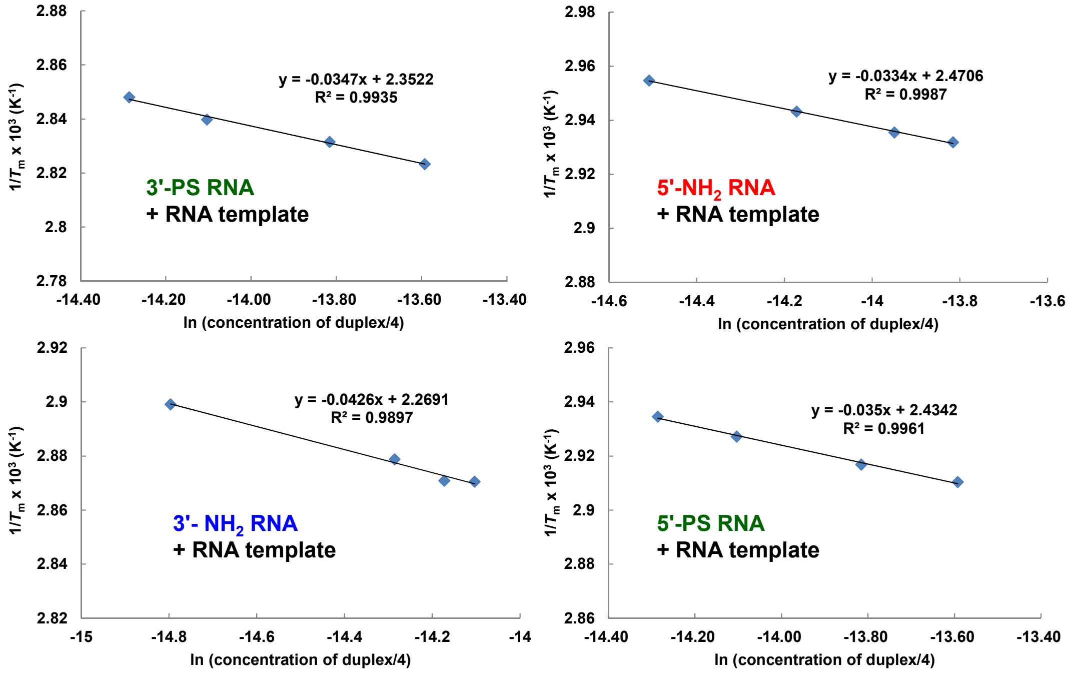

(B) Thermodynamic parameters of RNA.

	ΔH (kJ mol⁻¹)	ΔS (J K⁻¹mol⁻¹)	ΔG_{297} (kJ mol⁻¹)	K ($\times 10^{12}$ M⁻¹)	the rate of duplex formation[a]	the rate of ternary complex
3'-PS RNA + RNA template	-239.6	-563.6	-71.7	5.86	100	99.9
5'-NH_2 RNA + RNA template	-248.9	-615.0	-65.7	5.32	99.9	
3'- NH_2 RNA + RNA template	-195.2	-442.9	-63.2	0.18	99.8	99.8
5'-PS RNA + RNA template	-237.6	-578.3	-68.1	1.45	100	

Figure S7. Melting temperature measurement of RNA. All measurements were done in 20 mM Tris-HCl buffer (pH 7.2) containing 1.5-5 μM duplex and 10 mM $MgCl_2$ in 20 mM HEPES buffer (pH7.0). Reaction was monitored absorbance at 260 nm. (A) Plots of 1/Tm versus ln(concentration of duplex/4). The fitting curve (black line) was analyzed by linear regression as simple first-order kinetics by Microsoft Excel. (B) Thermodynamic parameters. The standard deviations for ΔH and ΔS were estimated from van't Hoff equation and (A).

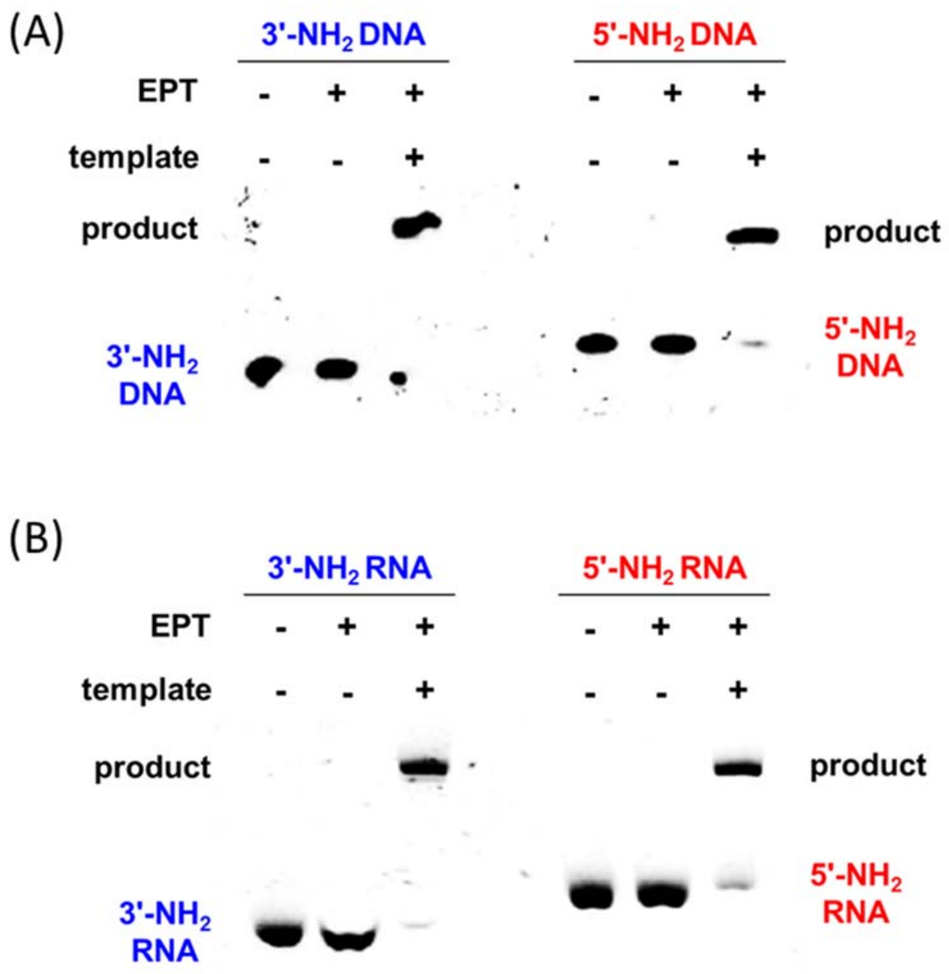

Figure S8 Chemical ligation reaction without template oligonucleotide.

~~PAGE (5.6 M urea, 25% formamide, 1×TBE) analysis of~~ T~~t~~he reactions were performed in a 12 μL reaction volume, containing each ~~2~~ 4 μM of EPT DNA (RNA), ~~4~~ 2 μM of ~~5'-~~NH_2- DNA (RNA) w/o 4 μM of template in 20 mM phosphate buffer (pH 8.0) containing 10 mM $MgCl_2$ at 25 °C. After 2 h, 12 μL loading buffer (80% formamide, 10 mM EDTA) was added to the reaction mixture, and 10 μL of aliquots were analyzed by electrophoresis on 15% denature polyacrylamide gel (5.6 M urea, 25% formamide, 1 × TBE) and visualized by scanning on a BioRad Molecular Imager FX (BioRad). No ligation occurred without the template.

A) Chemical ligation reaction between 3' or 5'-NH_2 DNA and 5' or 3'-EPT DNA with or without template DNA.

B) Chemical ligation reaction between 3' or 5'-NH_2 RNA and 5' or 2' -*O*Me-3'-EPT RNA with or without template RNA.

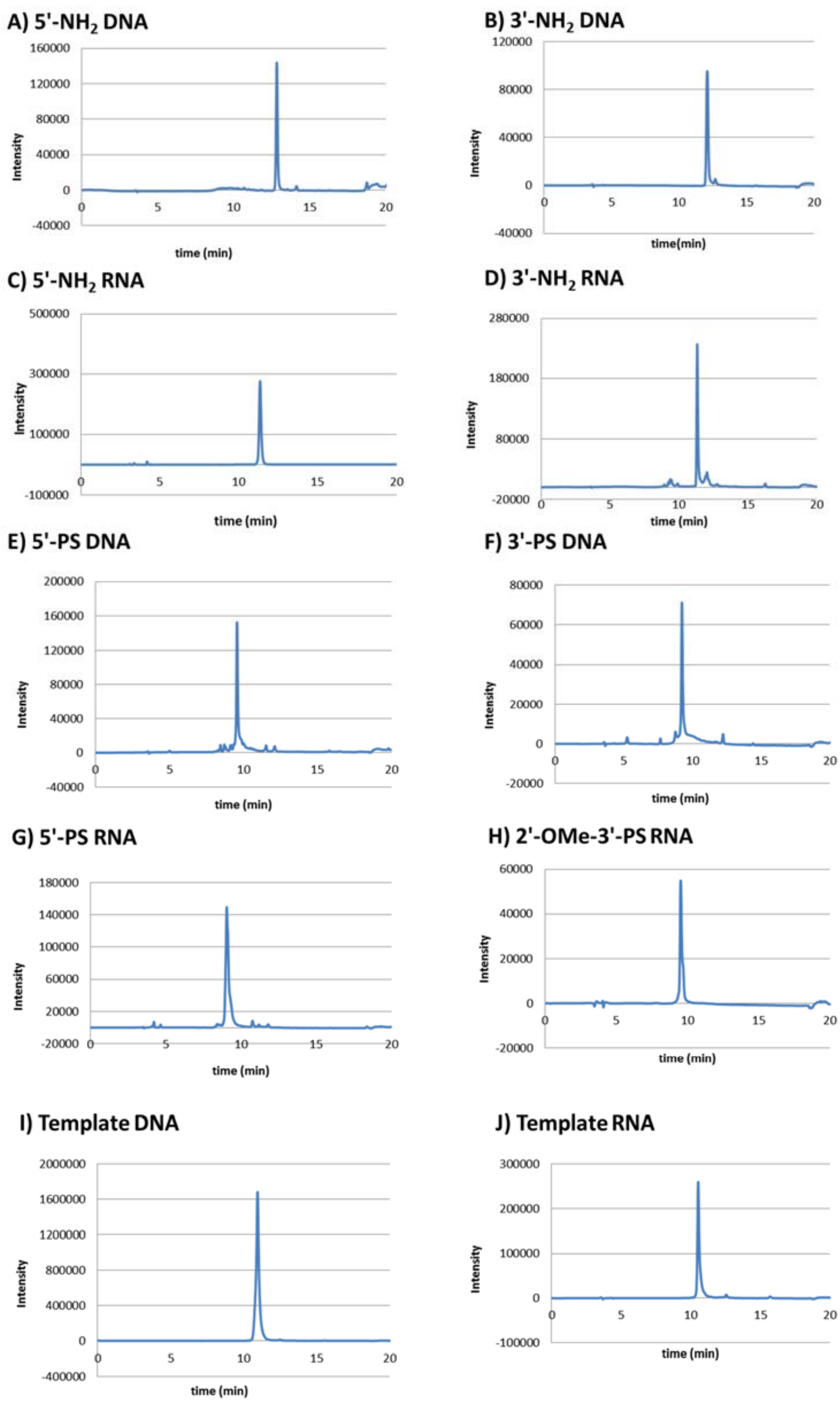

Figure S9. HPLC analysis of the ONs used in this study. The samples were analyzed by reversed-phase HPLC, using a Hydrosphere C18 column (4.6 × 250 mm; YMC). Eluent A was 5% acetonitrile (MeCN) in 50 mM triethylammonium acetate (TEAA) buffer (pH 7.0), and eluent B was 100% MeCN. The concentration of eluent B was increased from 5–30% over 15 min for A, B, I, J or, 10-25% over 15min for C or 5–40% over 15 min for D~ H at a flow rate of 1.0 mL/min. Absorption was monitored at 260 nm.

Sample	Sequences	Mw	
		calcd	found
Ligation Product from 3'-EPT DNA and 5'-NH_2 DNA	5'-d(GCTGAAGGGC) 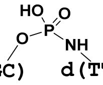 d(TTTTGAACTCTGC)-FAM-3'	7638.1 $(M + H)^+$	7638.1 $(M + H)^+$
Ligation Product from 5'-EPT DNA and 3'-NH_2 DNA	5'-FAM-d(GCTGAAGGGC) 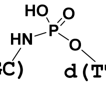 d(TTTTGAACTCTGC)-3'	7638.1 $(M + H)^+$	7638.9 $(M + H)^+$
Ligation Product from 2'-OMe-3'-EPT RNA and 5'-NH_2 RNA	5'-r(GCUGAAGGGC$_{OMe}$) 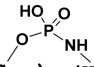 r(UUUUGAACUCUGC)-FAM-3'	7919.9 $(M - H)^-$	7919.3 $(M - H)^-$
Ligation Product from 5'-EPT RNA and 3'-NH_2 RNA	5'-FAM-r(GCUGAAGGGC) 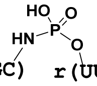 r(UUUUGAACUCUGC)-3'	7875.9 $(M - H)^-$	7875.4 $(M - H)^-$

Table S3: MALDI-TOF MS analysis of ligation products

Mass data were obtained using ultraflex III MALDI-TOF mass spectrometer (Bruker Daltonics) by positive or negative mode using 3-hydroxypicolinic acid (HPA) and Diammmonium hydrogen citrate (DHC) as matrixes. The ligation products were isolated by isopropanol precipitation (with NaOAc), polyacrylamide gel electrophoresis (7.5M urea, 15% formamide, 1×TBE) the following gel extraction (crash and soak methods) and desalting with NAP-10.

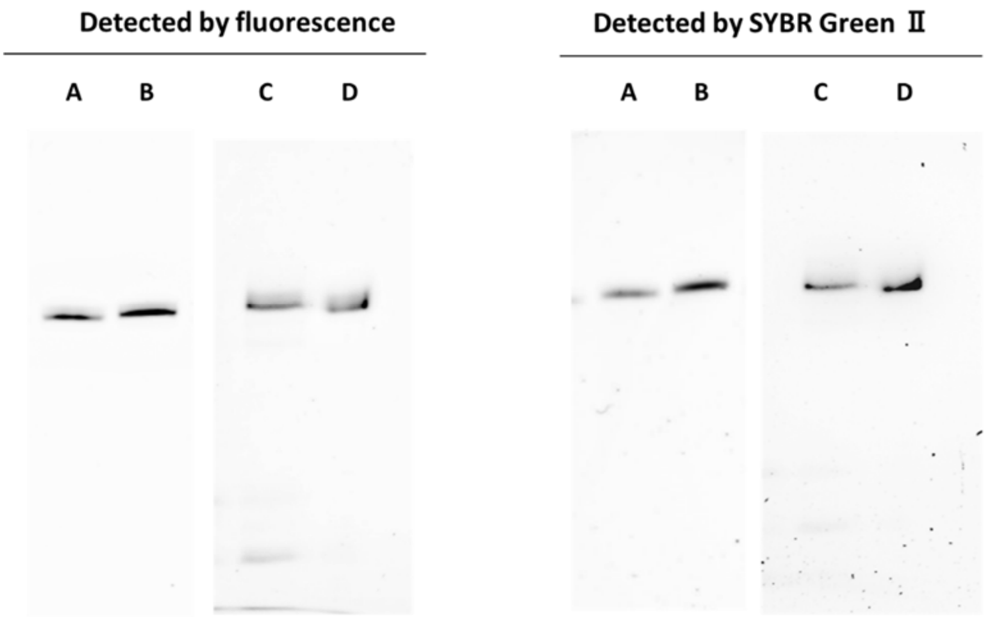

Figure S10. PAGE analysis of ligation products isolated by gel extraction (Products in the main text). Samples: ligation product from; A) 3'-NH_2 DNA and 5'-EPT DNA, B) 5'-NH_2 DNA and 3'-EPT DNA, C) 3'-NH_2 RNA and 5'-EPT RNA, D) 5'-NH_2 RNA and 2'-*O*Me-3'-EPT RNA. The samples were analyzed by 15% denaturing polyacrylamide gel (7.5 M urea, 25% formamide, 1×TBE, 25W for 1hr 22min for DNA ligation products, and 20W for 1hr 52 min for RNA ligation products) with detection by Fluorescence and SYBR Green II.

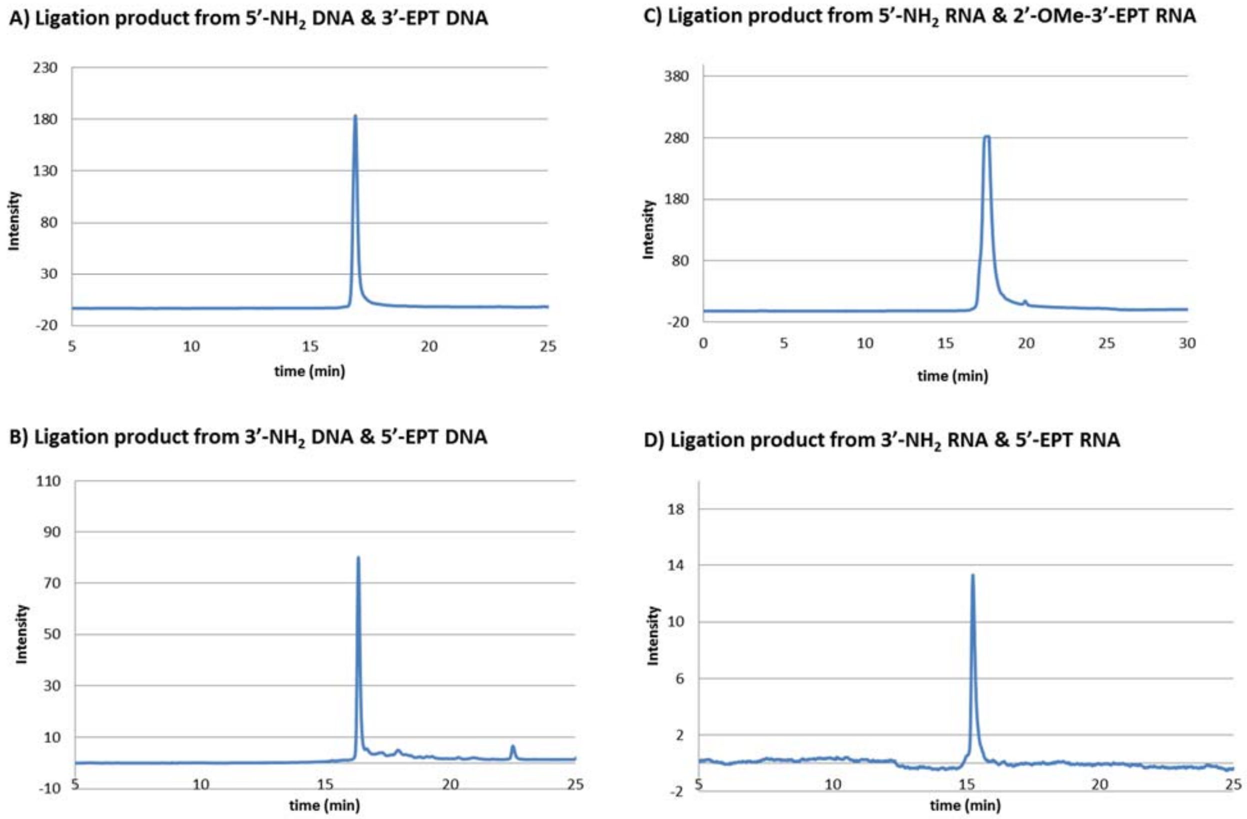

Figure S11. HPLC analysis of the ligation products isolated by gel extraction. Ligation product from; A) 5'-NH_2 DNA & 3'-EPT DNA, B) 3'-NH_2 DNA & 5'-EPT DNA, C) Ligation product from 5'-NH_2 RNA & 2'-*O*Me-3'-EPT RNA, D) Ligation product from 3'-NH_2 RNA & 5'-EPT RNA: The isolated products were analyzed by reversed-phase HPLC, using a Hydrosphere C18 column (4.6 × 250 mm; YMC). Eluent A was 5% acetonitrile (MeCN) in 50 mM triethylammonium acetate (TEAA) buffer (pH 7.0), and eluent B was 100% MeCN. The concentration of eluent B was increased from 5–40% over 20 min, at a flow rate of 1.0 mL/min. Fluorescence was monitored at 518 nm with excitation at 492 nm. For HPLC analysis of B) and D), the elution was performed at 60 ºC.

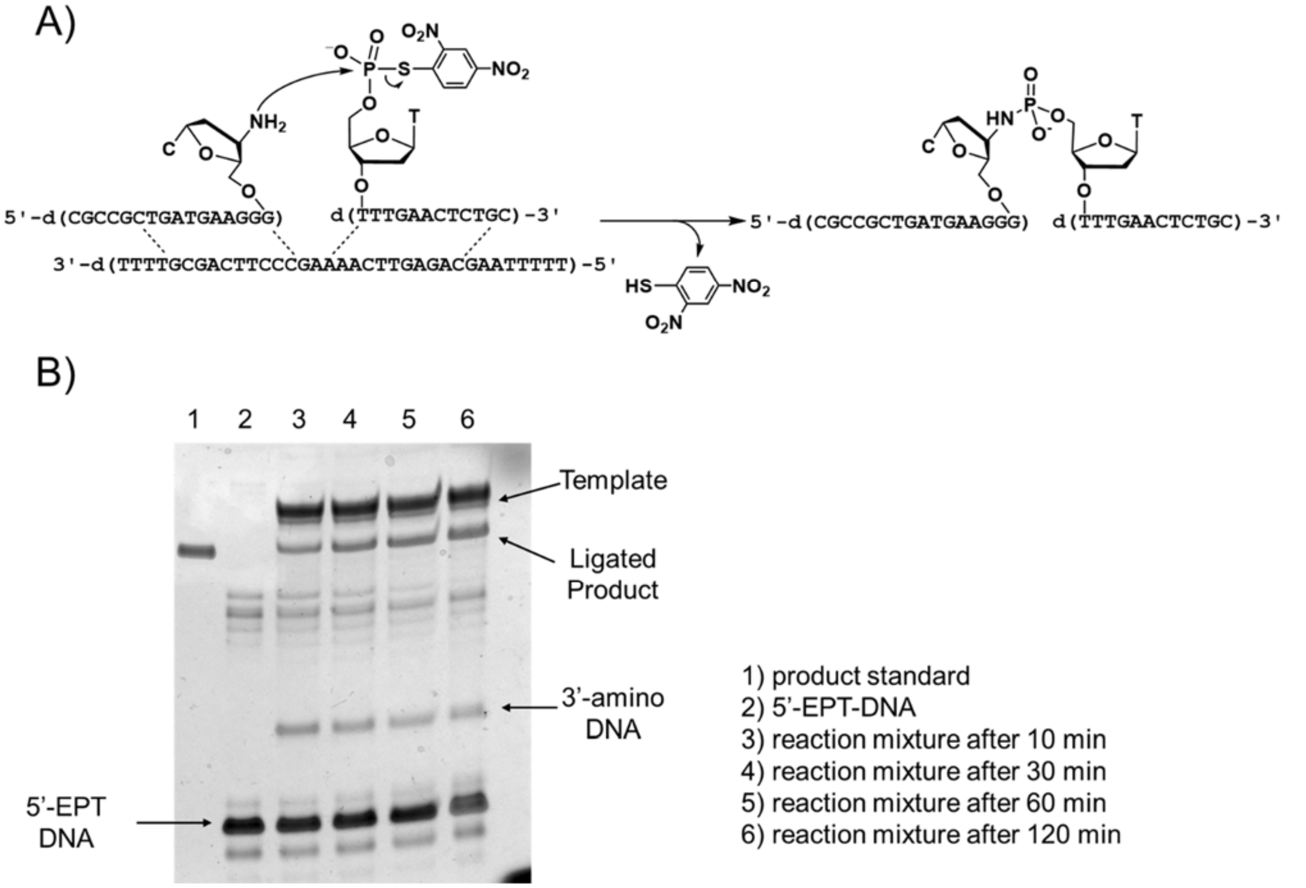

Figure S12 Ligation with non-fluorescence-labelled amino-DNA without isolation of EPT-DNA. A) Reaction scheme: 5'-EPT-DNA was obtained by the reaction of 5'-PS DNA with DNFB (DNFB 40 mM, 5'-PS DNA 214 µM, 1 hr, sodium tetraborate 40 mM) and the following isopropanol precipitation. Reaction conditions for ligation; EPT-DNA 20 µM, amino-DNA 5 µM, template DNA 10 µM, sodium tetraborate 50 mM, $MgCl_2$ 10 mM.

B) PAGE-analysis of the ligation reaction (15% denaturing PAGE; 7.5M urea, 1xTBE, stained by Stains-All). The product standard is a native-type DNA having same sequence as that of the ligated product. The ligation yield was calculated to be 78% after 120 min reaction based on the relative band intensity of the ligation product and the product standard. The several weak bands are trace impurities formed in PS-DNA synthesis or EPT-DNA synthesis, such as phosphorothioate dimer and 5'-phosphate DNA.

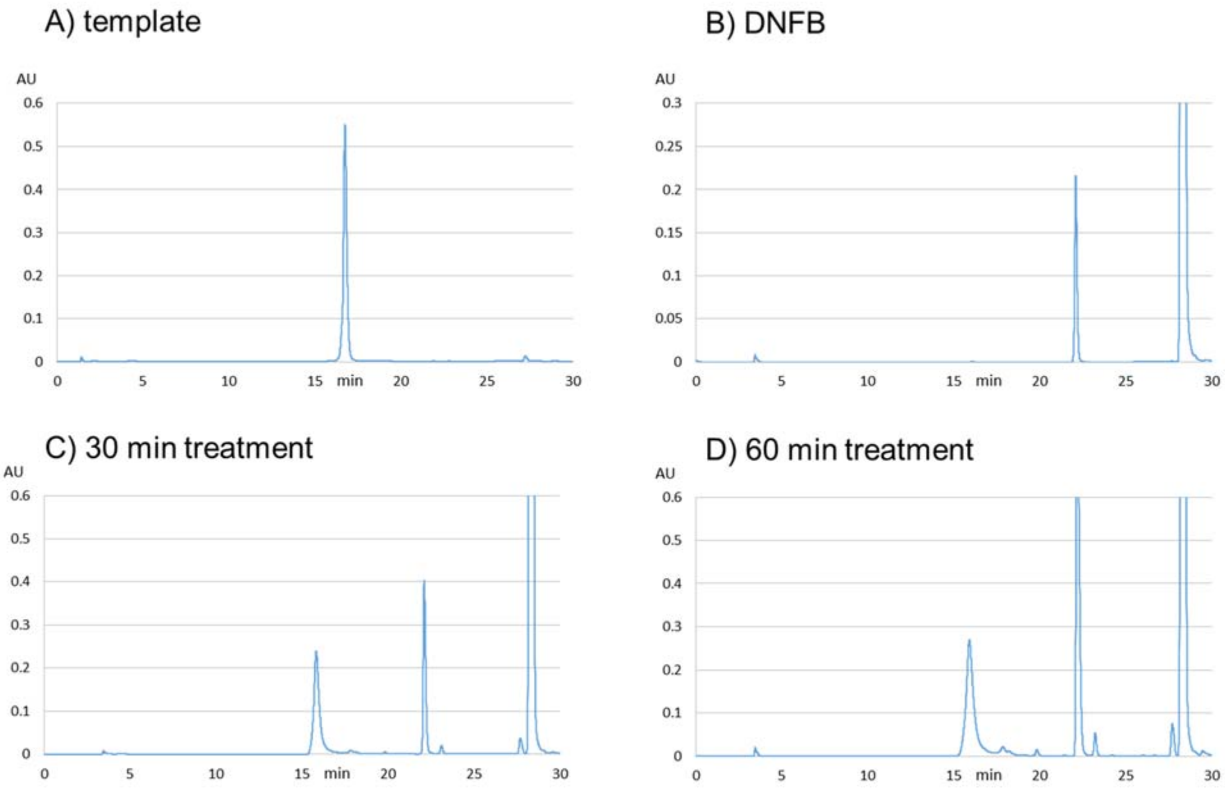

Figure S13: HPLC analysis of DNA template after treatment of DNFB. Conditions: DNA template 50 μM, 50 mM sodium tetraborate with 40% DMSO, 40 mM DNFB, 25 ºC. A) DNA template, B) DNFB, C) Template after 30 min treatment with DNFB. D) Template after 60 min treatment with DNFB. New peaks of template-derived species was observed, and no chemical modification was confirmed by MALDI-MS analysis ([M+H]+ calcd: 10708.81, found; 10705.11). These results suggest that no side-reaction would occur for oligonucleotides.

Sequence of the DNA template: 5'-d(TTTTTAAGCAGAGTTCAAAAGCCCTTCAGCGTTTT)-3'

HPLC analysis was performed using: hydrosphere C18 column (4.6 x 250 mm; YMC). Eluent A was 5% acetonitrile (MeCN) in 50 mM triethylammonium acetate (TEAA) buffer (pH 7.0), and eluent B was 100% MeCN. The concentration of eluent B was increased from 0–40% over 20 min, at a flow rate of 1.0 mL/min. Absorption was monitored at 260 nm.

A)

Sample	Sequences	Mw calcd	Mw found
5'-PS DNA	HO O HS-P-O d(TTTTGAACTCTGC)-3'	4011.63 (M + H)$^+$	4012.18 (M + H)$^+$
3'-NH_2 DNA	5'-d(CGCCGCTGATGAAGGGC) NH_2	5234.94 (M + H)$^+$	5232.79 (M + H)$^+$
Template	5'-d(TTTTTAAGCAGAGTTCAAAAGCCCTTCAGCGTTTT)-3'	10708.80 (M + H)$^+$	10707.30 (M + H)$^+$
Ligation Product	5'-d(CGCCGCTGATGAAGGGC) HO O HN-P-O d(TTTTGAACTCTGC)-3'	9212.58 (M + H)$^+$	9211.61 (M + H)$^+$

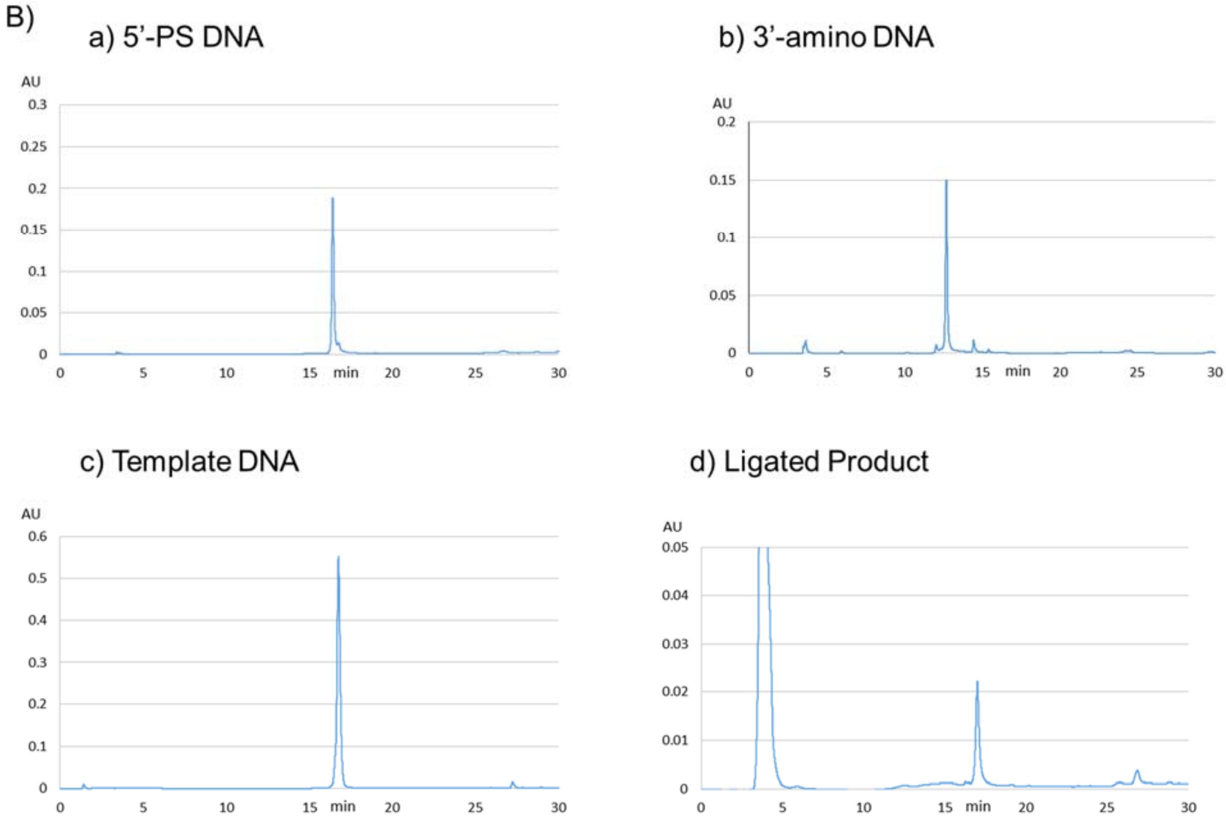

Figure S14. A) MALDI-MS data and B) HPLC analysis of substrates and ligation product without FAM label in the supporting information (Figure S12). The ligated product was isolated by PAGE and the following gel extraction (crash and soak method, desalting with NAP-10.). HPLC conditions: Hydrosphere C18 column (4.6 x 250 mm; YMC). Eluent A was 5% acetonitrile (MeCN) in 50 mM triethylammonium acetate (TEAA) buffer (pH 7.0), and eluent B was 100% MeCN. The concentration of eluent B was increased from 0–40% over 20 min with 260 nm UV detection. The peak around 16-17 min in d) is that of the ligated product. The peak around 4 min should be injection shock or that of non-oligonucleotide contamination from gel extraction.

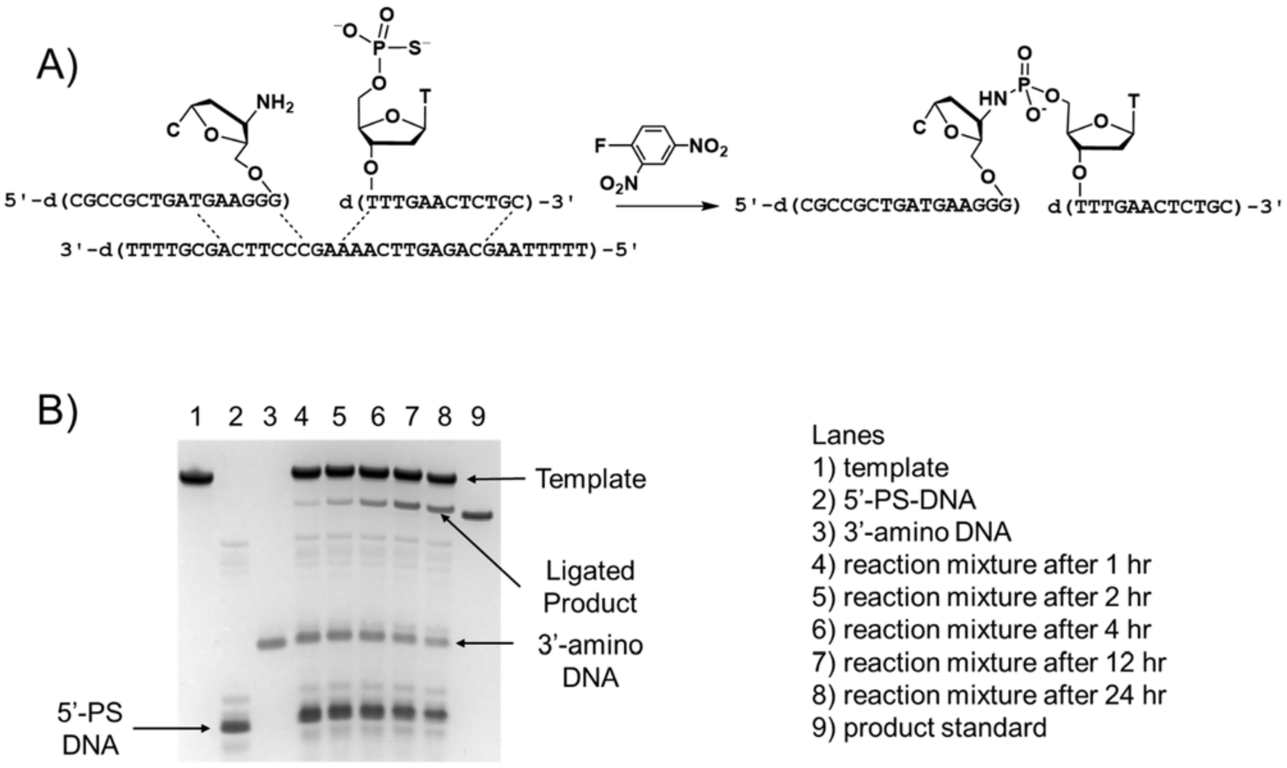

Figure S15. DNA ligation after annealing and the following *in situ* activation of 5'-PS DNA by DNFB. A) Reaction scheme. Reaction conditions; The reaction was performed in 50 µL volume, containing each 10 µM of 5'-PS DNA, 5 µM of 3'-amino DNA, 10 µM of template DNA and 500 µM of DNFB (40% DMSO) in 50 mM phosphate buffer (pH = 8), 10 mM $MgCl_2$, 100 mM NaCl. After the all the substrates and reagents except for DNFB was added, the mixture was heated up to 90 ºC for 3 min. After cooling down to 25 ºC over 30 min, the DNFB solution was added to the mixture and the reaction was started. B) Denaturing-PAGE analysis (5.6 M urea, 15% formamide, 1 x TBE, detected by Strains-All) of the reaction mixture after 1, 2, 4, 12, 24 hr. The ligation yield was calculated to be 52% after 12 hr based on the relative band intensity of the ligated product and the product standard. The product standard is the natural deoxynucleotide having the same sequence as the ligation product.

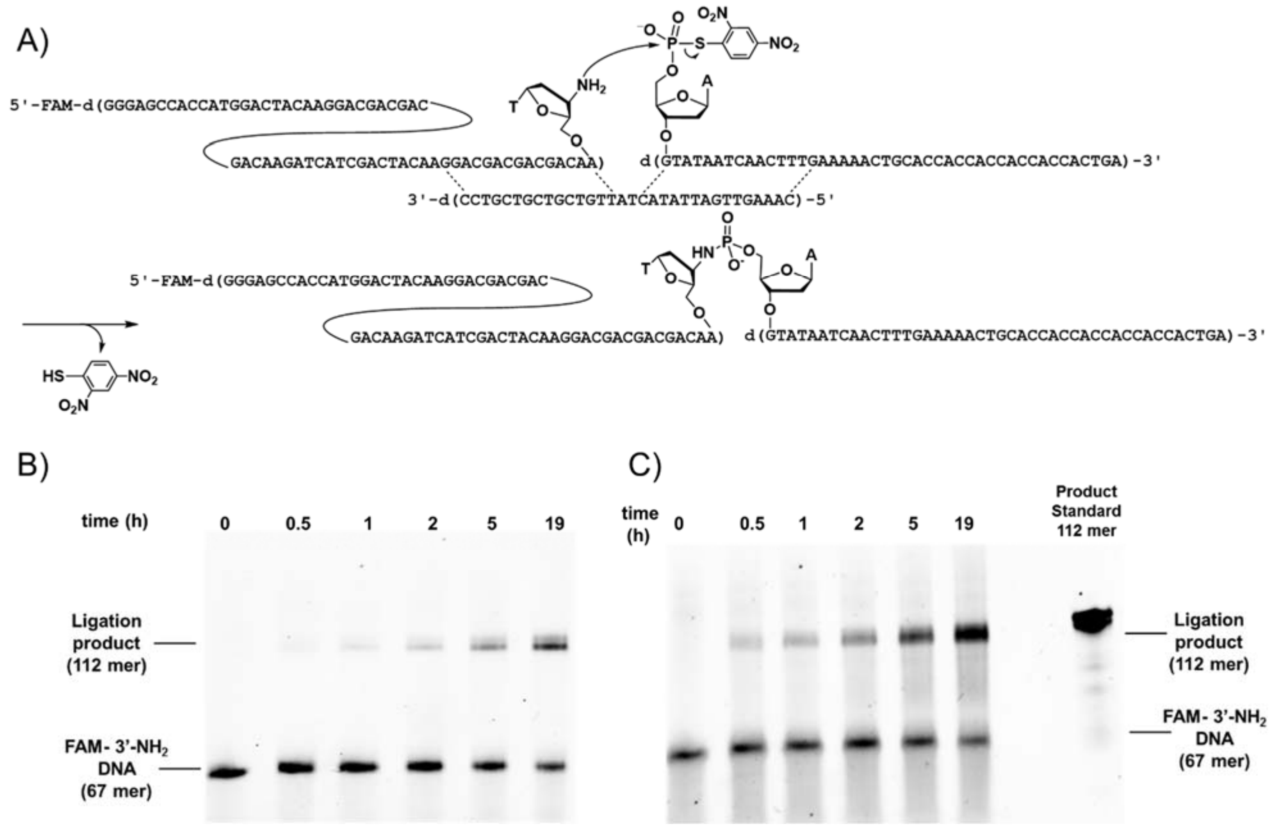

Figure S16. Ligation reaction with long deoxyoligonucleotides: (Amino-DNA: 67 mer, EPT-DNA: 45 mer) For FAM labelling, 6-FAM phosphoramidite (ChemGenes) was used in the automatic DNA synthesis. A) Reaction scheme. Reaction conditions; 4 μM of 5'-EPT DNA (activated by DNFB), 2 μM of 3'-NH_2 DNA, 4 μM of template DNA in 20 mM tetraborate buffer (pH 8.5), 10 mM $MgCl_2$ at 25°C. 5'-EPT-DNA was obtained by the reaction of 5'-PS DNA with DNFB (DNFB 30 mM, 5'-PS DNA 50 μM, 1 hr, sodium tetraborate (pH 8.5) 25 mM, 30% DMSO) and the following HPLC isolation (HPLC conditions; Hydrosphere C18 (4.6 x 250 mm; YMC) B conc. 5-40 % over 20 min. Solution A : 5% MeCN, 50 mM TEAA, Solution B : 100% MeCN Detection at 260nm). The reaction mixture was analyzed by 5% denaturing polyacrylamide gel (7.0 M urea, 1×TBE) after 0.5, 1, 2, 5, 19 hrs. B) PAGE gel result with FAM detection. The ligation yield was calculated to be 41% after 19 hr based on the relative fluorescence intensity of 3'-amino DNA and the ligation product. C) PAGE gel result with SYBR Green II detection. The product standard is the natural deoxyoligonucloetide having same sequence as the ligation product.

A)

Name	Sequences	Mw calcd	Mw found
long 3'-NH_2 DNA	5'-FAM-d(GGGAGCCACCATGGACTACAAGGACGACGACGAC AAGATCATCGACTACAAGGACGACGACGACAAT)-3' (NH_2)	21262.0 $(M + H)^+$	21260.7 $(M + H)^+$
long 5'-PS DNA	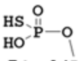 5'-d(AGTATAATCAACTTTGAAAAACTGCACCACCACCACCACCACTGA)-3'	13783.0 $(M + Na)^+$	13786.3 $(M + Na)^+$
long template DNA	5'-d(CCTGCTGCTGCTGTTATCATATTAGTTGAAAC)-3'	9781.4 $(M + H)^+$	9783.8 $(M + H)^+$

B)

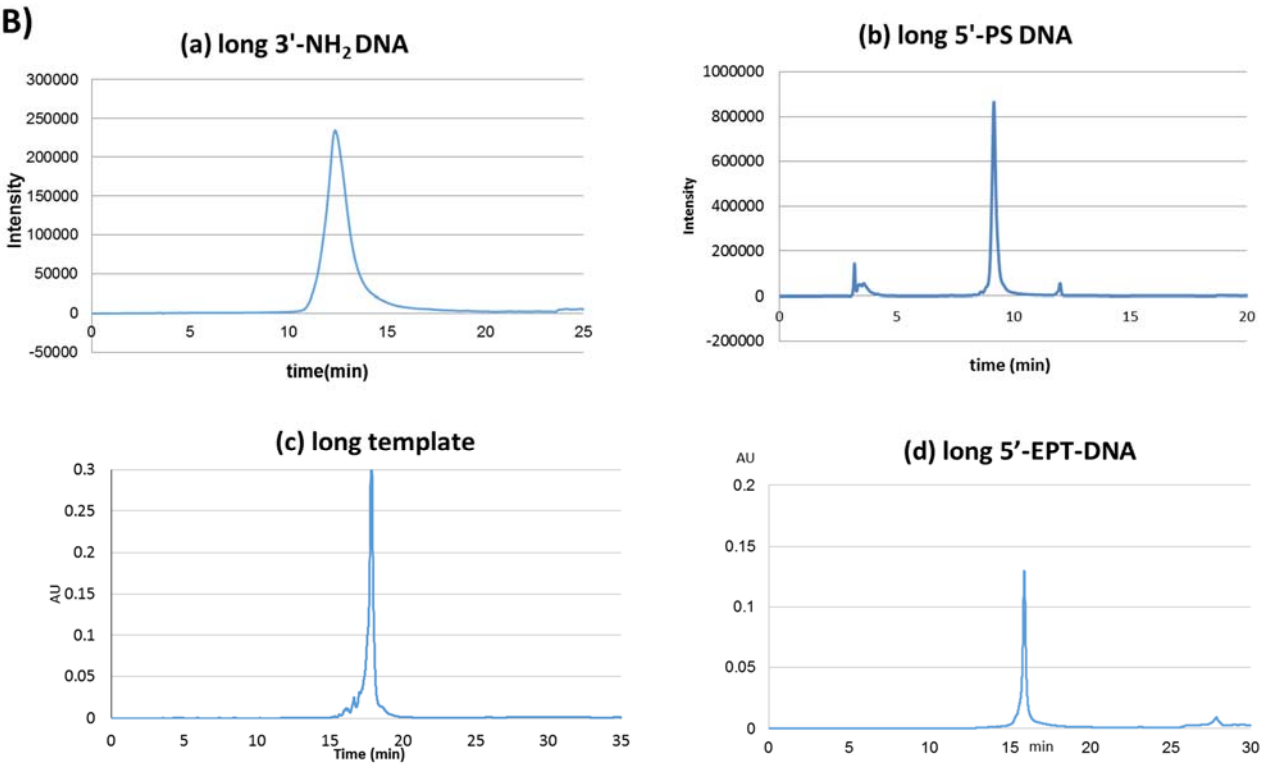

Figure S17. MALDI-MS and HPLC data of substrates for the ligation with long deoxyoligonucleotides. (Reaction in Figure S16) A) MALD-MS Data. TM (112 mer) peak cannot be observed even after the isolation from the PAGE gel, probably due to its large molecular weight. B) HPLC data of the substrates and the template. HPLC conditions for (a) ~ (c): Hydrosphere C18 column (4.6 x 250 mm; YMC). Eluent A was 5% acetonitrile (MeCN) in 50 mM triethylammonium acetate (TEAA) buffer (pH 7.0), and eluent B was 100% MeCN. The concentration of eluent B was increased from 5–40% over 20 min for amino-DNA, and 5–40% over 15 min for PS-DNA, and 0–40% over 20 min for template, at a flow rate of 1.0 mL/min. HPLC conditions for (d): Hydrosphere C18 column (10 x 250 mm; YMC), B) The concentration of eluent B was increased from 0 to 40% over 20 min at a flow rate of 3.0 mL/min. The UV absorbance was monitored at 260 nm.

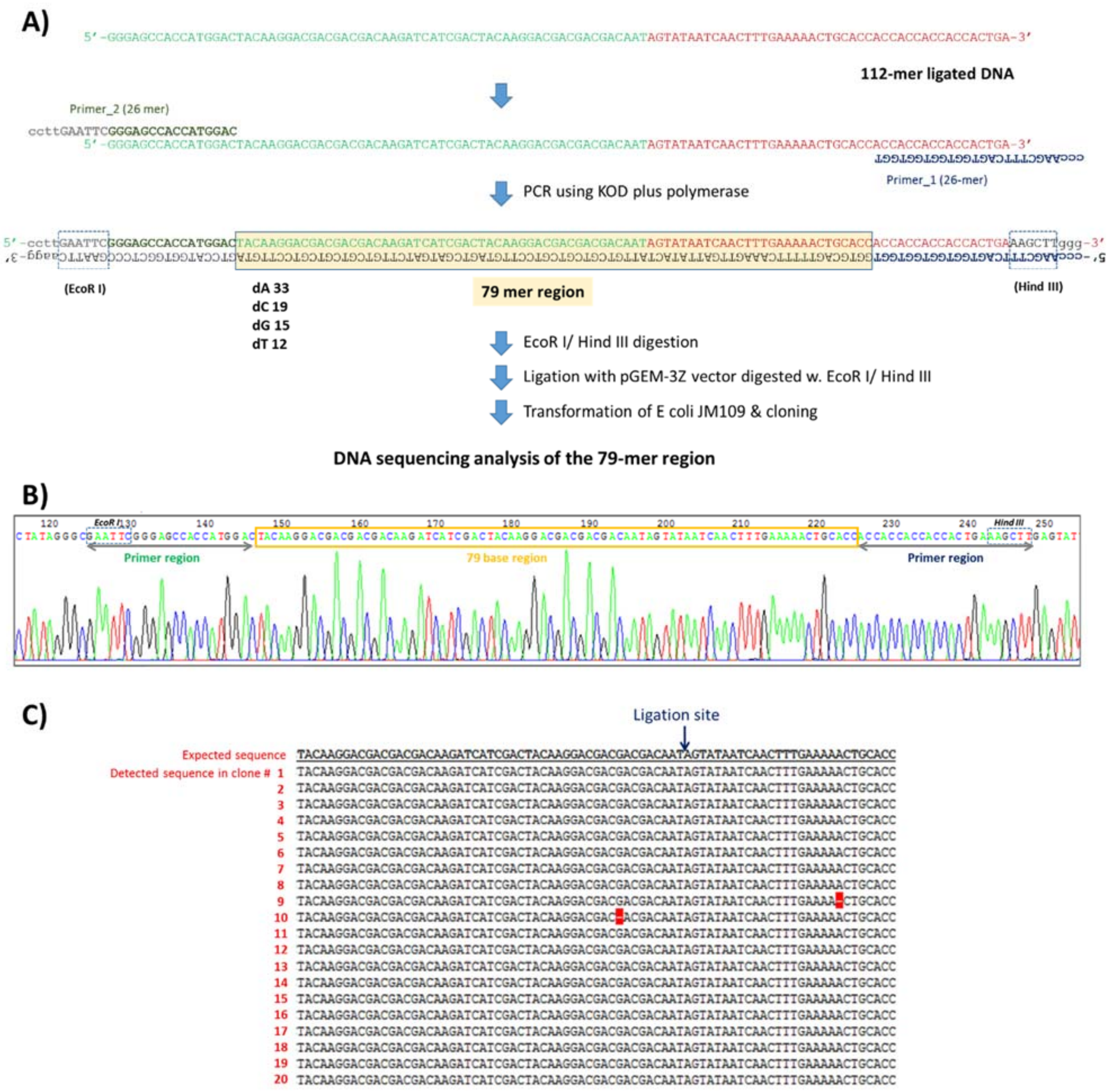

Figure S18. Sequence analysis of the ligation product from long deoxyoligonucleotides. A) A scheme for the DNA sequencing analysis. Sequence of 112-mer DNA, which was prepared by the chemical ligation (Fig. S16), was analyzed on ABI PRISM 3100 Genetic Analyzer (Applied Biosystems). The 112-mer ligated DNA was purified by denaturing PAGE and then used as a template in the PCR reaction. Composition of the PCR reaction mixture is as follows: 40 nM 112-mer DNA, 1 μM Primer_1, 1 μM Primer_2, 0.2 mM dNTPs, 0.01 U/μL KOD Plus. In the reaction, the sample was denatured at 94 °C for 10 s (2 min for the first cycle), annealed at 55 °C for 10 s and extended at 68 °C for 30 s (7.5 min for the last cycle) for 5 cycles on a 2720 Applied Biosystems Thermal Cycler. B) A representative chromatogram of the sequencing results. C) Aligned sequences of the 79-mer region of 20 clones tested in this assay. Red blank shows the deletion.

Procedures and analytical data for chemical synthesis of nucleotide derivatives

NMR spectra were recorded on JNM-AL300 (JEOL), JNM-AL-400, (JEOL) JNM-ECA-400 (JEOL) and JNM-ECA-500 (JEOL) spectrometers. Chemical shifts were reported in ppm on the δ scale relative to residual $CHCl_3$ (δ = 7.26 for ^{1}H NMR and δ = 77.0 for ^{13}C NMR) and 85% phosphoric acid (0.0 ppm) for ^{31}P NMR as an internal reference respectively. Mass spectra were recorded on JMS-T100LC (JEOL)

Synthetic protocols for compound **1** were reported[1,2].

Scheme S1 Synthesis of 5′-Amino Uridine Phosphoramidite unit 2[3]

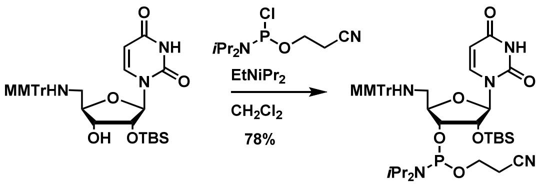

2

2′-*O*-(*tert*-Butyldimethylsilyl)-3′-*O*-[2-cyanoethoxy(*N*,*N*-diisopropylamino)phosphino]-5′-amino-(4-monomethoxytrityl)-5′-deoxyuridine (2)

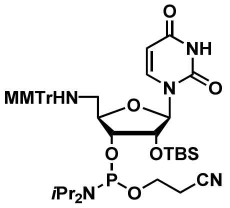

To a solution of 2′-*O*-(*tert*-butyldimethylsilyl)-5′-amino-(4-monomethoxytrityl)-5′-deoxyuridine (0.31 g, 0.50 mmol)[4] in CH_2Cl_2 (5.0 mL) was added *N*,*N*-diisopropylethylamine (0.21 mL, 1.2 mmol), 4-dimethylaminopyridine and 2-cyanoethyl-*N*,*N*-diisopropylchlorophosphoramidite (0.21 mL, 0.95 mmol) on ice, and the mixture was stirred at rt. After 2 h the mixture was partitioned between AcOEt and saturated *aq*. $NaHCO_3$ and the organic layer was washed with brine, dried (Na_2SO_4), filtered and concentrated *in vacuo*. The residue was purified by a neutral silica gel column chromatography, eluted with hexane-AcOEt (1/2, v/v), to give **2** (0.34 g, 0.39 mmol, 78%, as a white foam). ^{1}H NMR (400 MHz, $CDCl_3$) mixture of two diastereomers 7.98 (d, 1H, *J* = 8.4 Hz), 7.49-7.46 (m, 8H), 7.39-7.36 (m, 4H), 7.32-7.15 (m, 12H), 6.84 (d, 2H, *J* = 7.8 Hz), 6.82 (d, 2H, *J* = 7.8 Hz), 5.75-5.74 (m, 2H), 5.67 and 5.60 (2 d, 2H, *J* = 8.4 Hz), 4.36-4.31 (m, 3H), 4.24 (dd, 1H, *J* = 4.0, 1.6 Hz), 4.02-3.82 (m, 4H), 3.79 (2s, 6H), 3.79-3.45 (m, 7H), 2.90 and 2.72 (2 d, 2H, *J* = 11.8 Hz), 2.60 (t, 2H, *J* = 12.4 Hz), 2.32-2.24 (m, 3H), 1.16-1.09 (m, 18H), 1.03 (d, 6H, *J* = 6.8 Hz), 0.92 and 0.91 (2 s, 18H), 0.18, 0.16, 0.13 and 0.11 (4 s, 12H) ^{13}C NMR (100MHz, $CDCl_3$) mixture of two diastereomers 163.4, 163.2, 158.2, 158.1, 145.9, 145.8, 140.3, 140.0, 137.7, 137.7, 129.9, 129.9, 128.7, 128.6, 128.1, 126.7, 126.6, 117.7, 117.4, 113.3, 102.3, 102.0, 91.1, 90.9, 82.4, 81.6, 75.0, 73.6, 73.4, 71.6, 71.5, 70.4, 70.4, 58.4, 58.2, 58.0, 57.7, 55.3, 45.7, 44.6, 43.4, 43.2, 43.1, 25.9, 25.8, 24.8, 20.6, 20.5, 20.1, 18.2, -4.6, -4.7 ^{31}P NMR (161 MHz, $CDCl_3$) δ 135.71, 134.72.; ESIMS-LR *m/z* 852 [(M + Na)$^{+}$]; ESIMS-HR calcd for $C_{44}H_{60}N_5NaO_7PSi$ 852.3897, Found 852.3892.

Scheme S2 Synthesis of 3′-Amino Cytidine CPG unit 6[4]

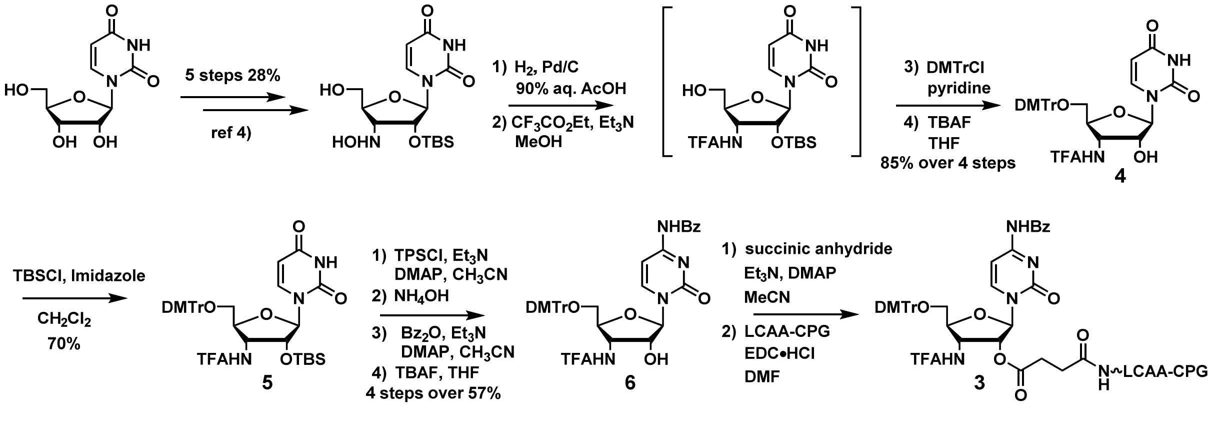

5′-*O*-(4,4′-Dimethoxytrityl)- 3′-(trifluoroacetamido)-3′-deoxyuridine (4)

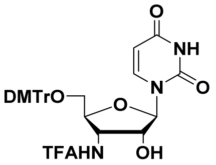

To a solution of 2′-*O*-(*tert*-butyldimethylsilyl)-3′-(hydroxyamino)-3′-deoxyuridine[4] (0.56 g, 1.51 mmol) in 90% *aq*. AcOH (15 mL) was added Pd/C (10%, 0.12 g) and stirre d under atmospheric pressure of H_2 at rt for 2 h. The insolubles were filtered off thr ough a Celite pad and the filtrate was concentrated *in vacuo*. The residue was coeva porated with AcOEt and dried under reduced pressure. The resulting reside was dissolved in MeOH (15 mL) and added triethylamine (0.63 mL, 4.53 mmol) and ethyl trifluoroacetate (0.54 mL, 4.53 mmol). The mixture was stirred at rt for 16 h and the solvent was concentrated *in vacuo*. The residue was coevaporat ed with pyridine and dissolved in pyridine (15 mL). The mixture was added DMTrCl (0.77 g, 2.27 mmo l) and stirred at rt. After stirring for 14 h, the reaction was quenched by addition of MeOH and the reac tion mixture was partitioned between AcOEt and H_2O and the organic layer was washed with saturate d *aq*. $NaHCO_3$ and brine, dried (Na_2SO_4), filtered and concentrated *in vacuo*. The residue was coevap orated with toluene and the resulting reside was dried under reduced pressure. Finally, to a solution of th e residue in THF (15 mL) was added TBAF (1.0 M solusion in THF, 1.66 mL, 1.66 mmol) at 0 °C and stirred at rt for 1 h. The reaction mixture and partitioned between AcOEt and H_2O. The organic layer was washed with saturated *aq*. $NaHCO_3$ and brine, dried (Na_2SO_4), filtered and concentrated *in vacu o*. The residue was purified by a silica gel column chromatography, eluted with hexane-AcOEt (1/2, v/v), to give **4** (0.82 g, 1.28 mmol, 85%, as a white foam). ^{1}H NMR (400 MHz, $CDCl_3$) δ 10.70 (br s, 1H, -NH), 8.34-8.32 (d, 1H, H-6, *J* = 8.2 Hz), 7.743 (br s, 1H, -NHTFA), 7.42-7.21 (m, 9H, Ar), 6.85-6.82 (m, 4H, Ar), 6.26 (br s, 1H, 2′-OH), 5.81 (s, 1H, H-1′), 5.27-5.25 (d, 1H, H-5, *J* = 8.2 Hz), 4.94-4.88 (dt, 1H, H-3′, *J* = 9.2, 4.8 Hz), 4.50-4.49 (d, 1H, H-2′, *J* = 4.1 Hz), 4.33-4.30 (d, 1H, H-4′, *J* = 10.1 H z), 3.79(1) and 3.78(7) (each s, each 3H, -OMe), 3.59-3.56 (dd, 1H, H-5′, *J* = 11.9, 1.8 Hz), 3.47-3.44 (dd, 1H, H-5′, *J* = 11.9, 1.8 Hz), ^{13}C NMR (100 MHz, $CDCl_3$) δ 164.4, 158.8, 158.8, 157.9, 157.5, 150. 9, 144.2, 140.9, 135.0, 134.8, 130.4, 130.2, 128.2, 127.3, 117.2, 114.3, 113.5, 113.4, 102.3, 92.1, 87.4, 81. 3, 74.9, 59.8, 55.4, 48.9; ESIMS-LR *m/z* 640 [(M - H)$^-$]; ESIMS-HR calcd for $C_{32}H_{29}N_3F_3O_8$ 640.1907, Found 640.1919.

2′-*O*-(*tert*-Butyldimethylsilyl)-5′-*O*-(4,4′-dimethoxytrityl)- 3′-(trifluoroacetamido)-3′-deoxyuridine (5)

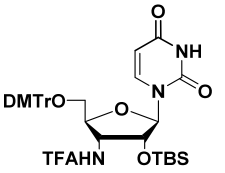

To a solution of **4** (0.30 g, 0.47 mmol) in CH_2Cl_2 (2.4 mL) was added imidazole (64 mg, 0.94 mmol), TBSCl (0.11 g, 0.71 mmol) and the mixture was stirred at rt. After 20 h, additional amount of imidazole (64 mg, 0.94 mmol), TBSCl (0.11 g, 0.71 mmol) and 4-dimethylaminopyridine (12 mg, 0.094 mmol) was added, and the mixture was stirred at rt. for further 3 h. Then the mixture was quenched by adding methanol and the residue was partitioned between AcOEt and H_2O. The organic layer was washed with saturated *aq*. $NaHCO_3$ and brine, dried (Na_2SO_4), filtered and concentrated *in vacuo*. The residue was purified by a silica gel column chromatography, eluted with hexane-AcOEt (2/1, v/v), to give **5** (0.25 g, 0.33 mmol, 70%, as a white foam). ^{1}H NMR (400 MHz, $CDCl_3$) δ 9.57 (br s, 1H, -NH), 8.13-8.10 (d, 1H, H-6, *J* = 8.2 Hz), 7.43-7.41 (m, 2H, Ar), 7.36-7.22 (m, 7H, Ar), 6.86-6.83 (m, 4H, Ar), 6.72-6.70 (d, 1H, -NHTFA, *J* = 8.7 Hz), 5.87(0)-5.86(5) (d, 1H, H-1′, *J* = 1.8 Hz), 5.25-5.23 (d, 1H, H-5, *J* = 8.2 Hz), 4.89-4.83 (dt, 1H, H-3′, *J* = 8.7, 5.0 Hz), 4.38-4.37 (dd, 1H, H-2′, *J* = 5.0, 1.4 Hz), 4.15-4.12 (dt, 1H, H-4′, *J* = 8.7, 2.3 Hz), 3.79(1) and 3.78(6) (each s, each 3H, -OMe), 3.61-3.58 (dd, 1H, H-5′, *J* = 11.4, 1.8 Hz), 3.45-3.42 (dd, 1H, H-5′, *J* = 12.8, 2.3 Hz), 0.91 (s, 9H, -C$(CH_3)_3$), 0.24 and 0.14 (each s, each 3H, $-CH_3 \times 2$), ^{13}C NMR (100 MHz, $CDCl_3$) δ 163.5, 158.9, 158.8, 157.3, 156.9, 150.4, 144.1, 140.0, 134.9, 134.8, 130.4, 130.2, 128.22, 128.17, 127.3, 117.1, 114.2, 113.4, 102.4, 90.1, 87.5, 81.7, 76.0, 60.6, 55.3, 50.1, 25.7, 18.0, -4.5, -5.4; ESIMS-LR *m/z* 754 [$(M - H)^-$]; ESIMS-HR calcd for $C_{38}H_{43}F_3N_3O_8Si$ 754.2772, Found 754.2788.

***N*[4]-Benzoyl-5′-*O*-(4,4′-dimethoxytrityl)- 3′-(trifluoroacetamido)-3′-deoxycytidine (6)**

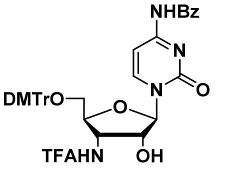

To a solution of **5** (76 mg, 0.10 mmol) in CH_3CN (2.0 mL) was added 4-dimethylaminopyridine (37 mg, 0.30 mmol), triethylamine (42 μL, 0.30 mmol) and 2,4,6-triisopropylbenzenesulfonyl chloride (91 mg, 0.30 mmol) on ice, and the mixture was stirred at rt for 2 h. Additional 4-dimethylaminopyridine (12 mg, 0.10 mmol), triethylamine (14 μL, 0.10 mmol) and 2,4,6-triisopropylbenzenesulfonyl chloride (30 mg, 0.10 mmol) were added and further stirred at rt for 1 h. Then the mixture was added 28% *aq*. NH_3 (2.0 mL) and kept at rt for 40 min. The reaction mixture was evaporated *in vacuo* and the residue was partitioned between AcOEt and H_2O. The organic layer was washed with saturated *aq*. $NaHCO_3$ and brine, dried (Na_2SO_4), filtered and concentrated *in vacuo*. The residue was dissolved in CH_3CN (2.0 mL) and added triethylamine (84 μL, 0.60 mmol), 4-dimethylaminopyridine (37 mg, 0.30 mmol) and benzoic anhydride (0.10 g, 0.45 mmol). After stirring at rt for 18 h, the reaction mixture was partitioned between AcOEt and H_2O and the organic layer was washed with saturated *aq*. $NaHCO_3$ and brine, dried (Na_2SO_4), filtered and concentrated *in vacuo*. The residue was dissolved in THF (1.0 mL) and added 1.0 M TBAF solution in THF (0.20 mL, 0.20 mmol), and the mixture was stirred at rt for 0.5 h. The reaction mixture purified by a silica gel column chromatography, eluted with hexane-AcOEt (1/3, v/v), to give **6** (42 mg, 0.057 mmol, as a white foam, 57%). ^{1}H NMR (400 MHz, $CDCl_3$) δ 8.95 (br s, 1H, -NHBz), 8.61-8.59 (d, 1H, H-6, *J* = 7.8 Hz), 7.90-7.88 (m, 2H. Ar), 7.61-7.57 (m, 1H, Ar), 7.50-7.46 (m, 2H, Ar), 7.43-7.41 (m, 2H, Ar), 7.33-7.23 (m, 9H, Ar, -NHTFA), 6.87-6.83 (m, 4H, Ar), 5.94 (s, 1H, H-1′), 4.85-4.79 (m, 1H, H-3′), 4.50-4.48 (d, 1H, H-2′, *J* = 5.5 Hz), 4.37-4.33 (dt, 1H, H-4′, *J* = 8.7, 2.3 Hz), 3.80(3) and 3.79(9) (each s, each 3H, -OMe), 3.62-3.58 (dd, 1H, H-5′, *J* = 11.4, 2.3 Hz), 3.45-3.41 (dd, 1H, H-5′, *J* = 11.4, 2.3 Hz), 2.04 (brs, 1H, 2′-OH), ^{13}C NMR (100 MHz, $CDCl_3$) δ 163.0, 158.8, 157.8, 157.4, 156.2,

145.0, 144.0, 135.4, 135.2, 133.3, 132.9, 130.3, 130.2, 129.1, 128.3, 128.2, 127.8, 127.3, 117.3, 114.4, 113.4, 97.3, 93.2, 87.4, 82.6, 75.1, 60.8, 55.3, 49.7

; ESIMS-LR *m/z* 767 [(M + Na)$^+$]; ESIMS-HR calcd for $C_{39}H_{35}F_3N_4NaO_8$ 767.2305, Found 767.2298.

N^4-Benzoyl-5′-*O*-(4,4′-Dimethoxytrityl)- 3′-(trifluoroacetamido)-3′-deoxycytidine LCAA-CPG unit (3)

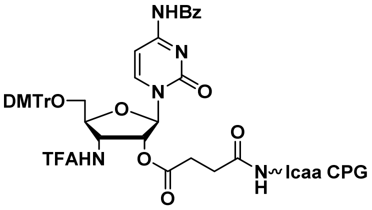

To a solution of **6** (35 mg, 0.050 mmol) in MeCN (1.0 mL) was added triethylamine (18 µL, 0.13 mmol), succinic anhydride (10 mg, 0.10 mmol) and 4-dimethylaminopyridine (1.2 mg, 0.010 mmol). The reaction mixture was stirred at rt for 1 h. Then the mixture was partitioned between 10% MeOH-$CHCl_3$ and H_2O and the organic layer was washed with saturated *aq*. $NaHCO_3$ and brine, dried (Na_2SO_4), filtered and concentrated *in vacuo*. The resulting residue was dissolved in DMF (1.0 mL) and added 1-ethyl-3-(3-dimethylaminopropyl) carbodiimide hydrochloride (12 mg, 0.060 mmol) and LCAA-CPG 1000 Å(150 mg, 85 µmol/g). The reaction mixture was shaken at rt for 24 h and the solid support was filtered and washed with DMF, MeOH and $CHCl_3$. The remaining amino groups were capped by treatment with Ac_2O (0.20 mL) in pyridine (1.8 mL). The resulting solid support was washed with DMF, MeOH and $CHCl_3$. The loading amount of **6** was estimated by DMTr cation assay (12 mg of solid support (**3**) was treated) to be 18 µmol/g (135 mg).

*DMTr cation assay

Solid support was treated with 50 mL of 60% $HClO_4$ (60% solution in water) in EtOH and absorbance at 498 nm (wavelength of maximum absorption of DMTr cation) was measured. Concentration of the solution was calculated with molar absorbance coefficient of DMTr cation (ε_{498} = 70000) and consequently the loading amount was found.

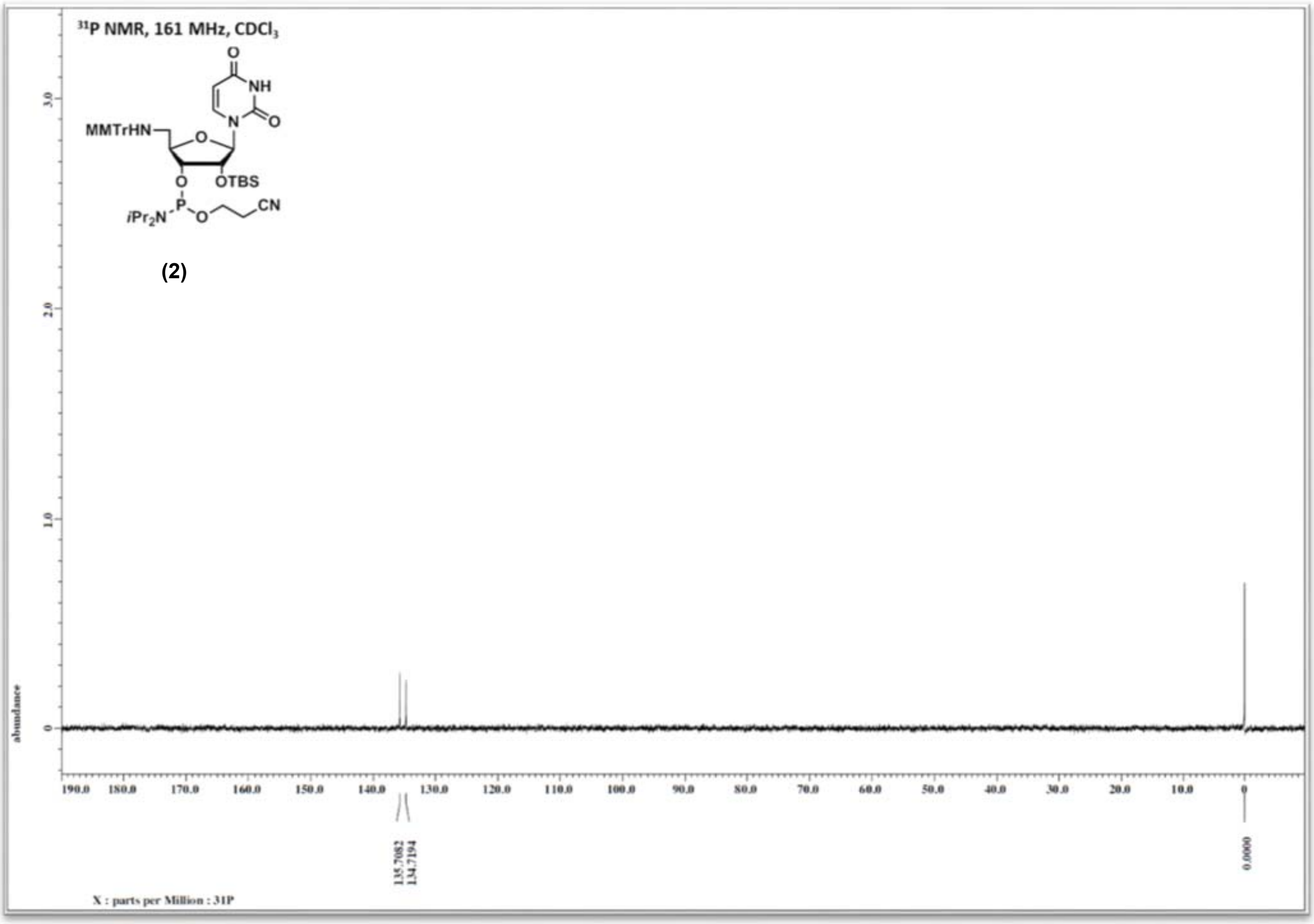

31P NMR, 161 MHz, CDCl3
MMTrHN
OTBS
iPr2N
CN
(2)
abundance
X : parts per Million : 31P
135.7082
134.7194
0.0000

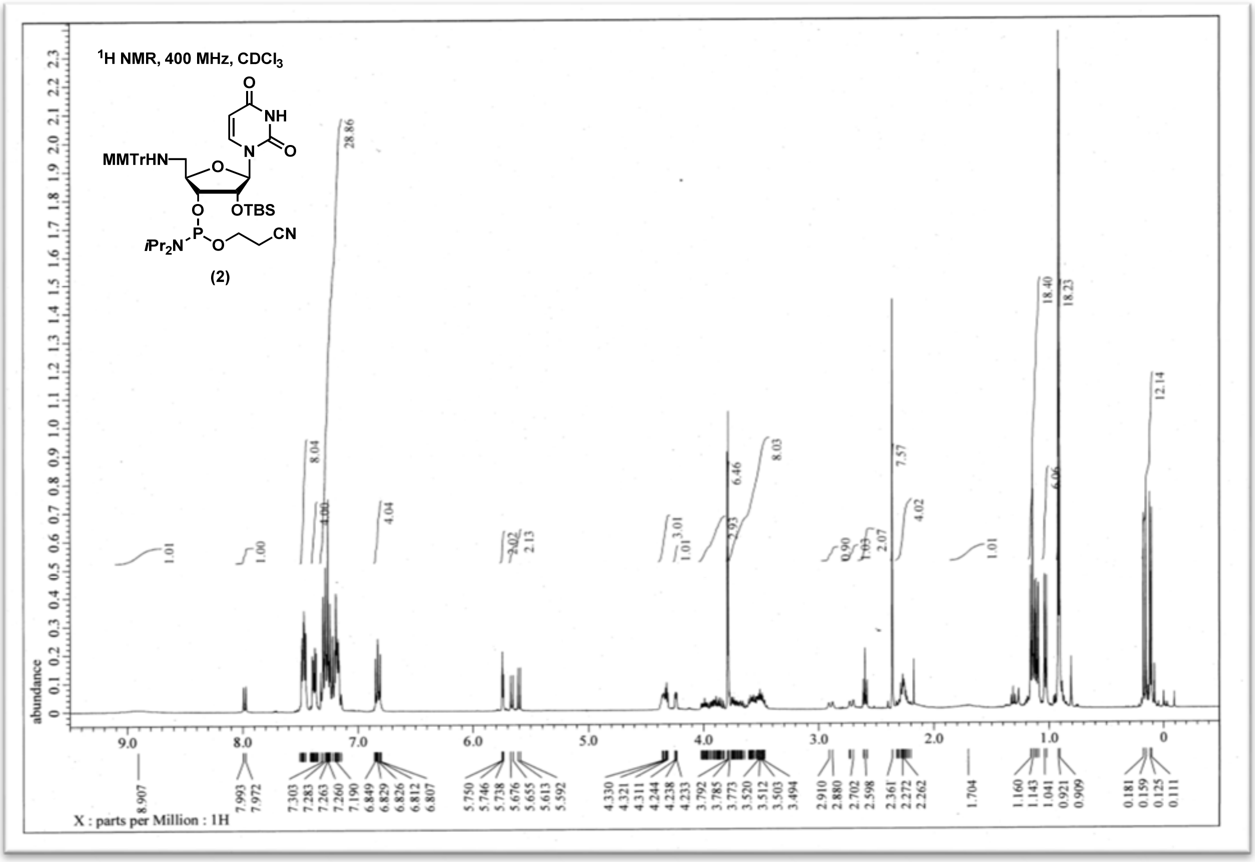
^{1}H NMR, 400 MHz, CDCl$_3$
MMTrHN
OTBS
iPr$_2$N
CN
(2)
X : parts per Million : 1H
abundance

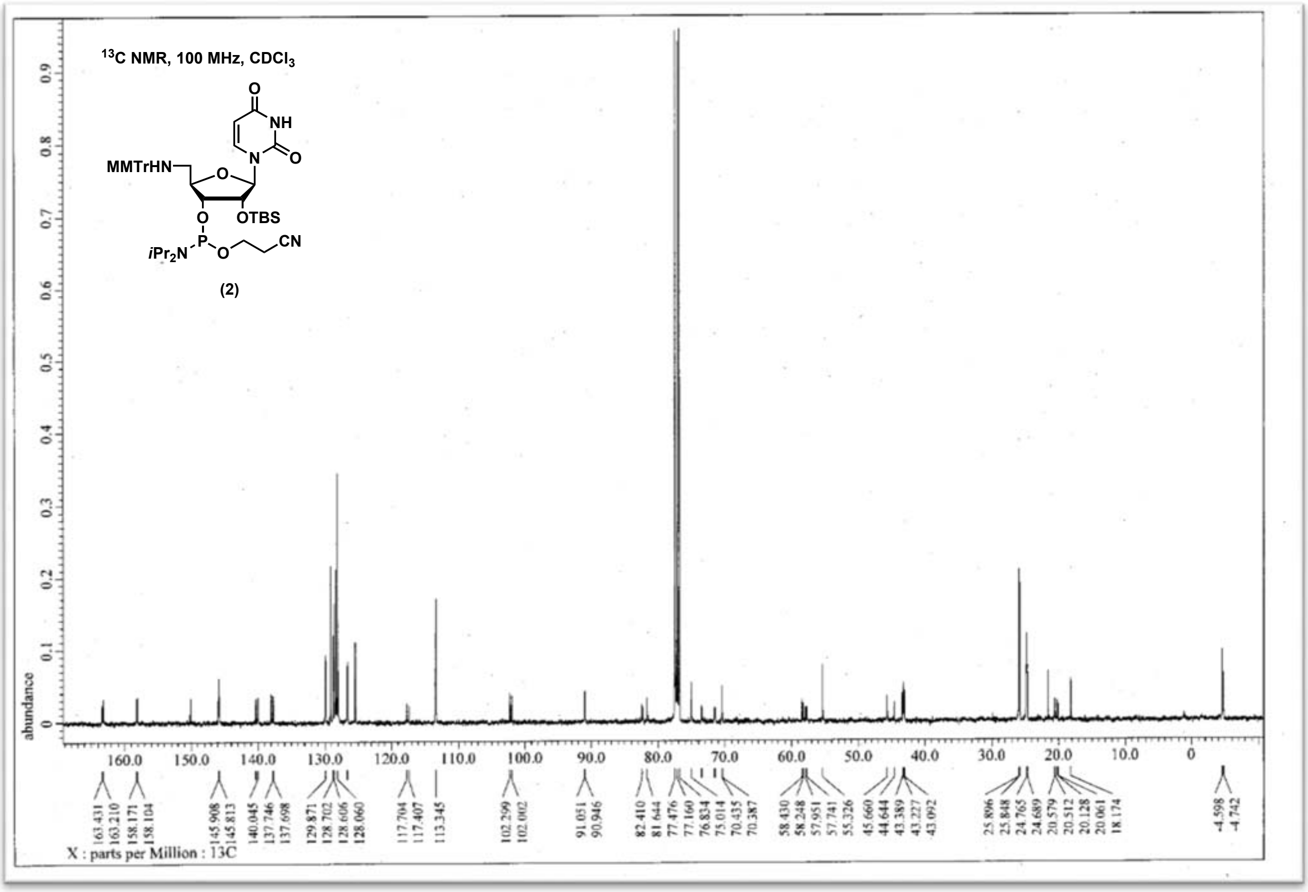
^{13}C NMR, 100 MHz, CDCl$_3$
MMTrHN
OTBS
iPr$_2$N
CN
(2)
X : parts per Million : 13C
abundance

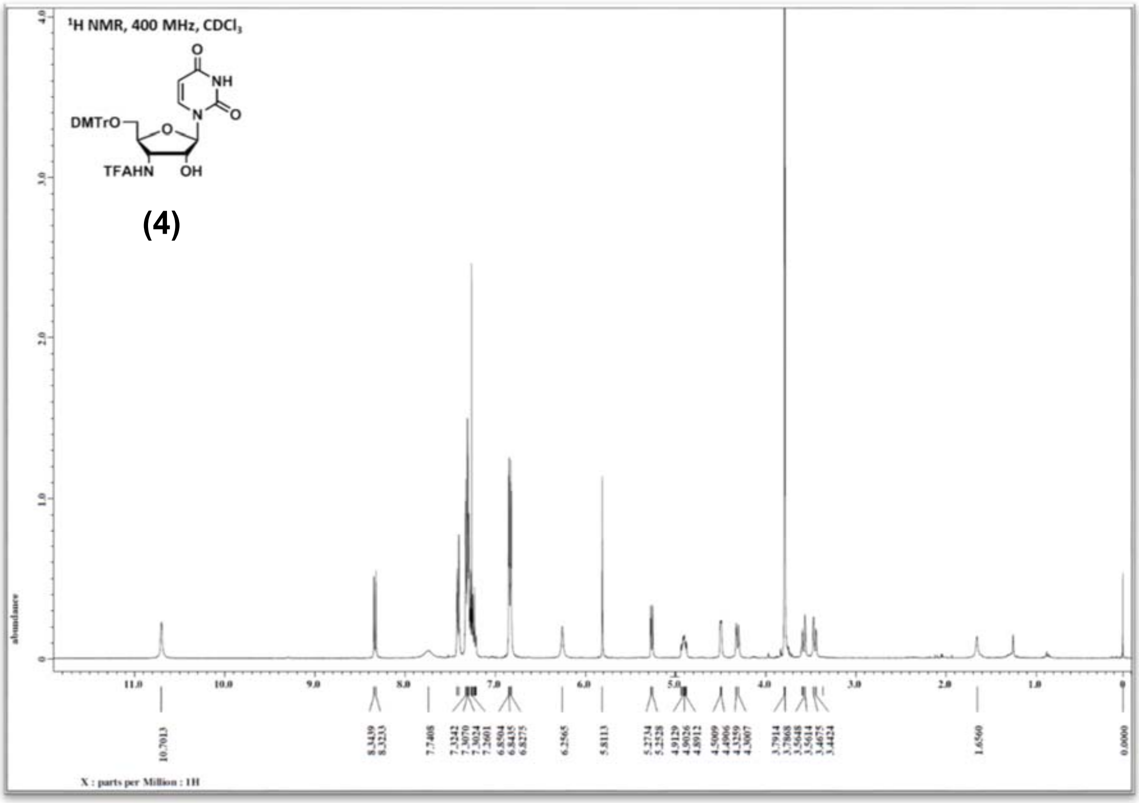

1H NMR, 400 MHz, CDCl3
DMTrO
TFAHN
OH
NH
(4)
X : parts per Million : 1H

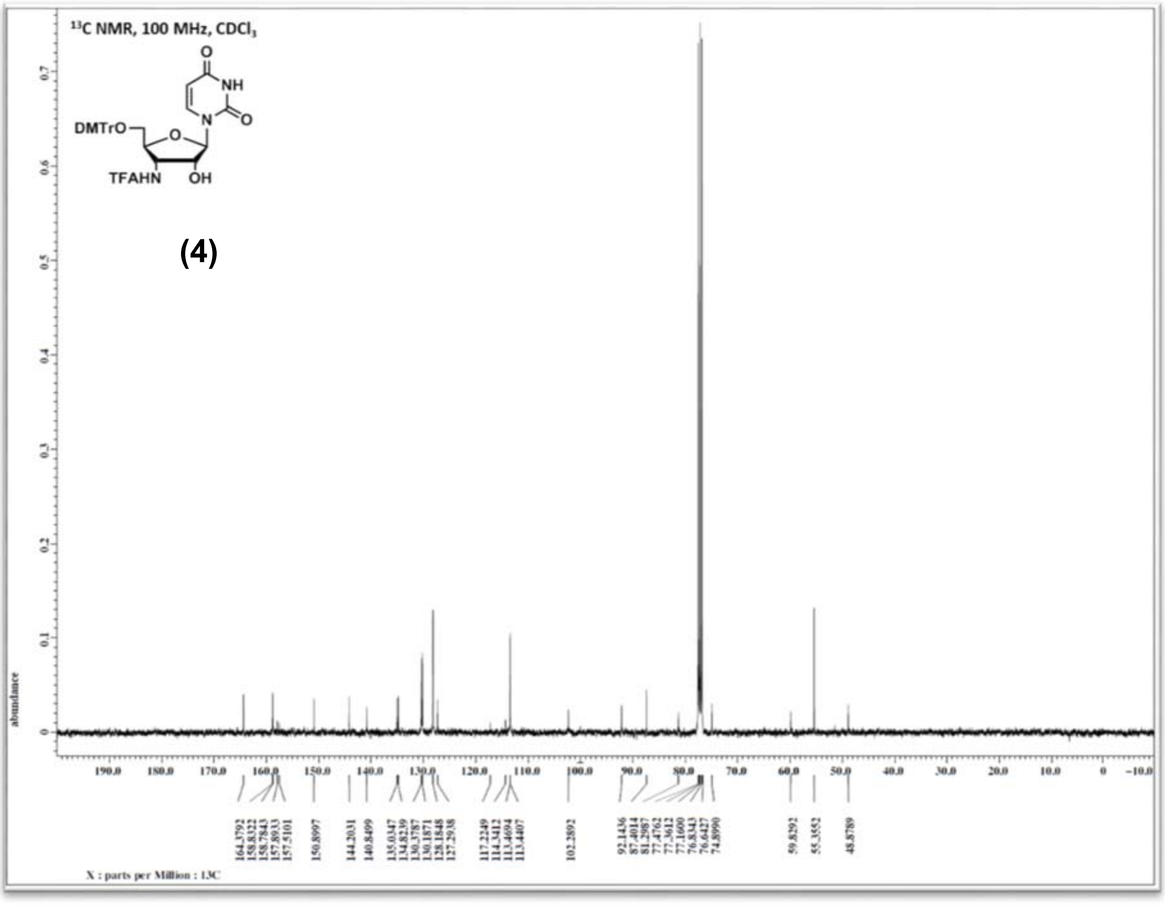

13C NMR, 100 MHz, CDCl3
DMTrO
TFAHN
OH
NH
(4)
X : parts per Million : 13C

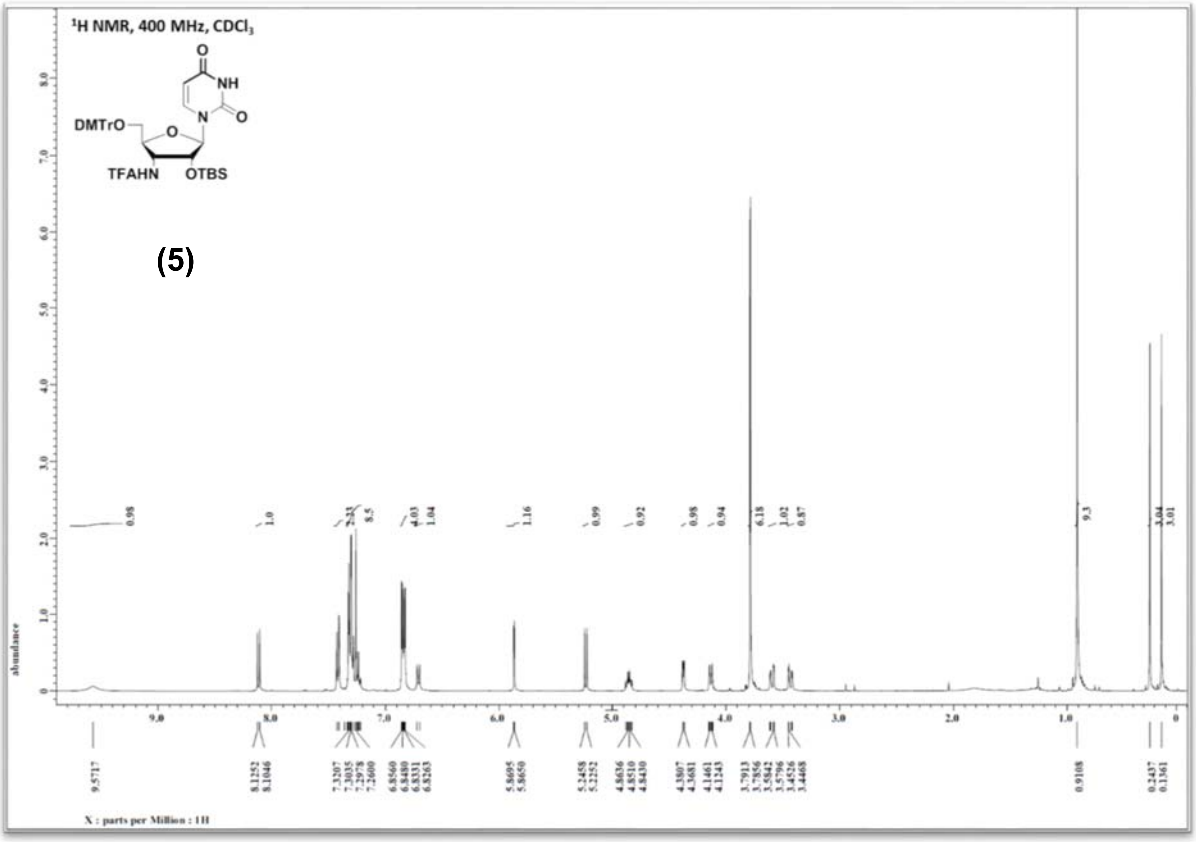
1H NMR, 400 MHz, CDCl3
DMTrO
TFAHN
OTBS
NH
(5)
abundance
X : parts per Million : 1H

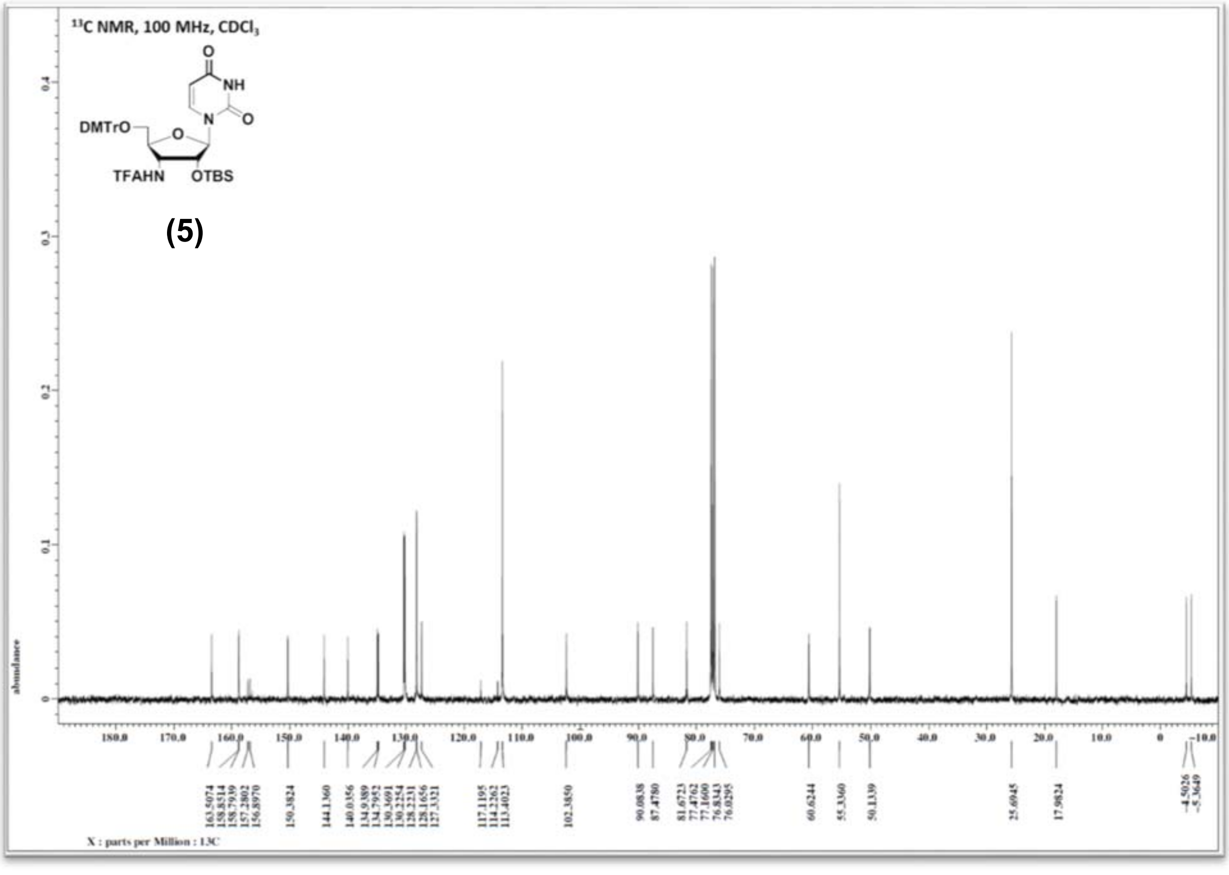
13C NMR, 100 MHz, CDCl3
DMTrO
TFAHN
OTBS
NH
(5)
abundance
X : parts per Million : 13C

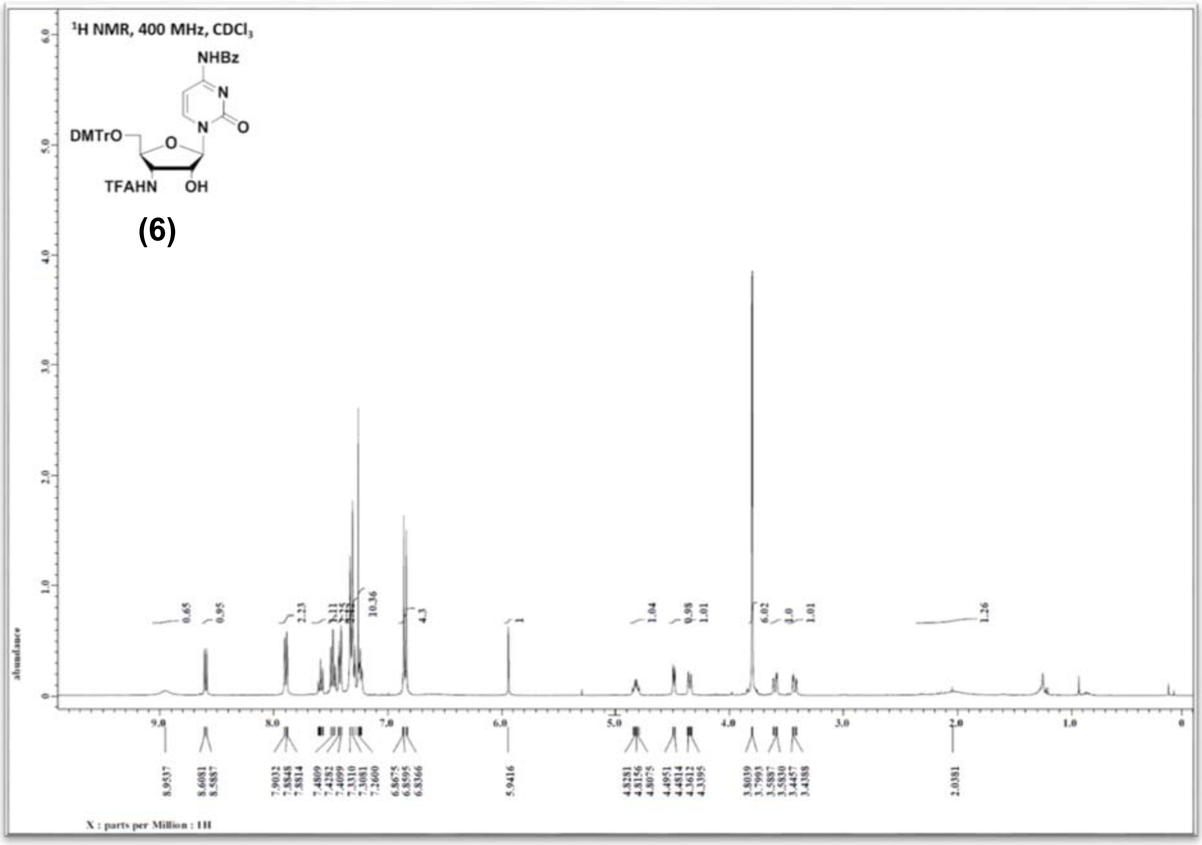

¹H NMR, 400 MHz, CDCl₃
NHBz
DMTrO
TFAHN
OH
(6)
X : parts per Million : 1H

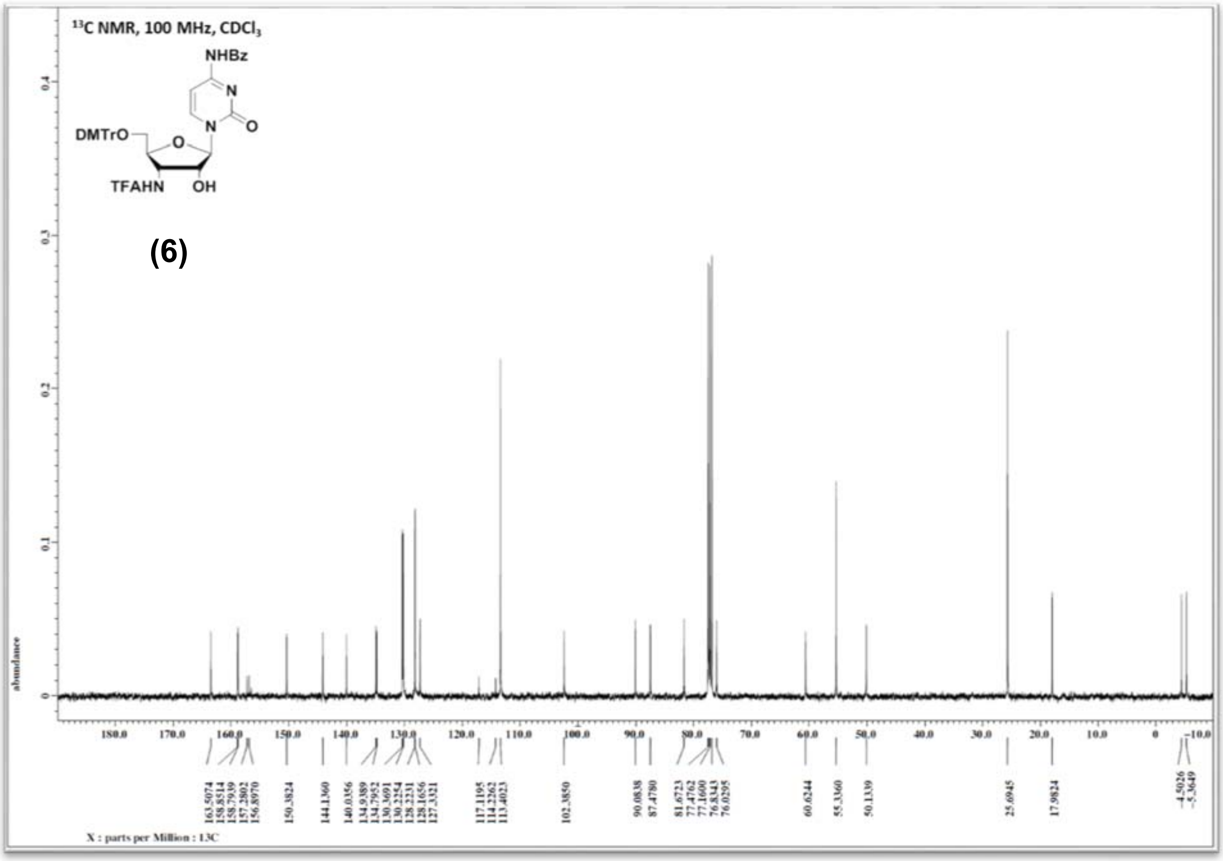

¹³C NMR, 100 MHz, CDCl₃
NHBz
DMTrO
TFAHN
OH
(6)
X : parts per Million : 13C

1. R. Eisenhuth and C. Richert, *J. Org. Chem.*, 2009, 74, 26-37.
2. J. S. Nelson, K. L. Fearon, M. Q. Nguyen, S. N. McCurdy, J. E. Frediani, M. F. Foy and B. L. Hirschbein, *J. Org. Chem.*, 1997, 62, 7278-7287.
3. I. Yamamoto, M. Sekine and T. Hata, *J. Chem. Soc., Perkin Trans. 1*, 1980, 306-310.
4. N. Kojima, I. E. Szabo and T. C. Bruice, *Tetrahedron*, 2002, 58, 867-879.
